# The Benzoylpiperidine Fragment as a Privileged Structure in Medicinal Chemistry: A Comprehensive Review

**DOI:** 10.3390/molecules29091930

**Published:** 2024-04-23

**Authors:** Giulia Bononi, Chiara Lonzi, Tiziano Tuccinardi, Filippo Minutolo, Carlotta Granchi

**Affiliations:** Department of Pharmacy, University of Pisa, Via Bonanno 6, 56126 Pisa, Italy; giulia.bononi@unipi.it (G.B.); c.lonzi2@studenti.unipi.it (C.L.); tiziano.tuccinardi@unipi.it (T.T.); filippo.minutolo@unipi.it (F.M.)

**Keywords:** benzoylpiperidine, phenyl(piperidin-4-yl)methanone, benzoylpiperidine-based small molecules, privileged structure, medicinal chemistry, bioisostere, drug design, therapeutic agents, diagnostic agents

## Abstract

The phenyl(piperidin-4-yl)methanone fragment (here referred to as the benzoylpiperidine fragment) is a privileged structure in the development of new drugs considering its presence in many bioactive small molecules with both therapeutic (such as anti-cancer, anti-psychotic, anti-thrombotic, anti-arrhythmic, anti-tubercular, anti-parasitic, anti-diabetic, and neuroprotective agents) and diagnostic properties. The benzoylpiperidine fragment is metabolically stable, and it is also considered a potential bioisostere of the piperazine ring, thus making it a feasible and reliable chemical frame to be exploited in drug design. Herein, we discuss the main therapeutic and diagnostic agents presenting the benzoylpiperidine motif in their structure, covering articles reported in the literature since 2000. A specific section is focused on the synthetic strategies adopted to obtain this versatile chemical portion.

## 1. Introduction

**Figure 1:**
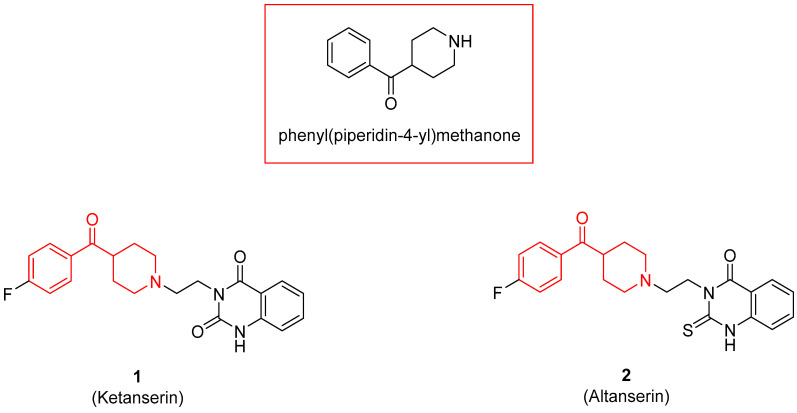
The benzoylpiperidine fragment (phenyl(piperdin-4-yl)methanone, in the red square) and the structures of two representative 5-HT_2A_ antagonists, ketanserin (**1**) and altanserin (**2**), with the benzoylpiperidine fragment highlighted in red.

The benzoylpiperidine fragment (phenyl(piperidin-4-yl)methanone, Figure 1) is an important chemical frame exploited in medicinal chemistry because it is present in many different bioactive compounds with a broad spectrum of therapeutic effects but also with diagnostic uses.

The piperidine ring can be considered a potential bioisostere of the piperazine ring, and when this bioisosteric replacement takes place, a good strategy to compensate for the role of the additional nitrogen atom of the piperazine is the introduction of a heteroatom to re-establish the loss of binding energy. In the case of benzoylpiperidine, this role is played by the carbonyl group, which is hopefully able to establish new interactions (i.e., hydrogen bonds) with the target [1]. The bioisosteric replacement of piperazine with the benzoylpiperidine moiety is therefore used in medicinal chemistry to evaluate the structure-activity relationship (SAR) of several classes of bioactive compounds, although this chemical modification does not always lead to an improvement in terms of biological activity [2,3,4,5,6,7].

Noteworthy, the benzoylpiperidine system is a privileged structure, together with 3-(piperidin-4-yl)benzo[*d*]isoxazole, in the design and development of atypical antipsychotic agents considering that this chemical frame is present in the structure of the two potent 5-HT_2A_ reference drugs ketanserin and altanserin (compounds **1** and **2**, Figure 1, see also Section 3.2.1) [1,8]. Moreover, it was found that the 4-(*p*-fluorobenzoyl)piperidine fragment present in both drugs is crucial for allowing the anchorage or the orientation of the ligand at the 5-HT_2A_ receptor, and the 4-(*p*-fluorobenzoyl)piperidine is also considered a butyrophenone pharmacophore constrained in a six-membered ring [9,10,11,12]. For these reasons, one of the most populated classes of therapeutic agents possessing the benzoylpiperidine fragment is that of serotoninergic and dopaminergic receptor ligands for the treatment of neuropsychiatric and neurodegenerative diseases (see Section 3.2.1).

The use of the benzoylpiperidine fragment in drug design also attracts the scientific community’s interest because of its quite easy and fast synthesis, which normally exploits safe and not too expensive reagents. Furthermore, from a structural point of view, the presence of a symmetry plane and the consequent lack of chiral centers in this fragment make this molecular unit a highly preferred structure to be inserted into candidate drugs since it excludes further issues concerning asymmetric synthesis, chiral separation, and biological assays on separate stereoisomers.

**Figure 2:**
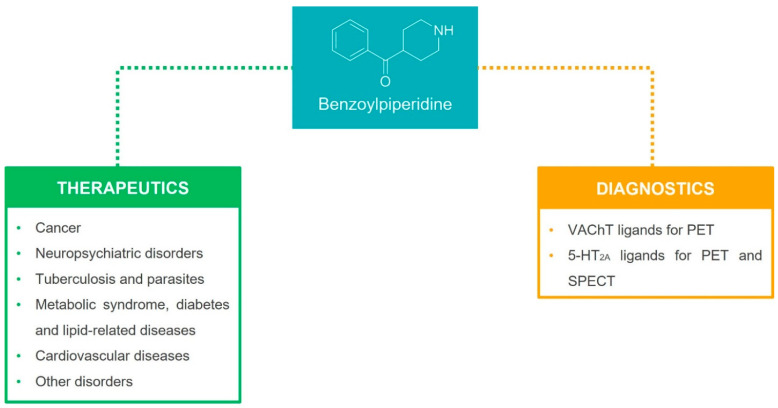
General classification of benzoylpiperidine-based small molecules as therapeutics and diagnostics.

In this review, we provide a general overview of therapeutic and diagnostic agents characterized by a benzoylpiperidine fragment, which have been reported in the literature since 2000 (Figure 2). The compounds discussed herein are classified on the basis of their therapeutic effects (anti-cancer, anti-psychotic, anti-thrombotic, anti-arrhythmic, anti-tubercular, anti-parasitic, anti-diabetic, neuroprotective agents, etc.) and then of their biological targets (Section 3.1, Section 3.2, Section 3.3, Section 3.4, Section 3.5 and Section 3.6). A separate section is dedicated to the benzoylpiperidine-based compounds used for diagnostic purposes (Section 3.7). Additionally, the most frequently used synthetic procedures to build this chemical fragment are described in Section 2.

**Figure 3:**
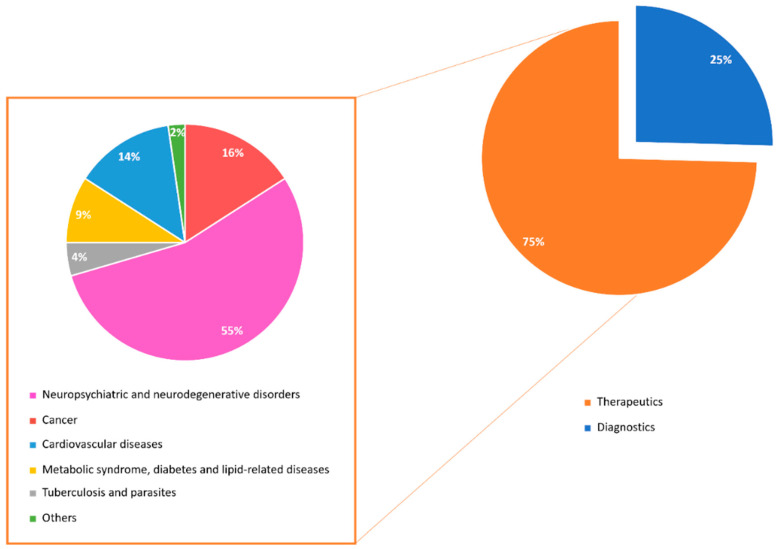
Percentages of therapeutic and diagnostic benzoylpiperidine-based molecules herein reported.

The analysis of the articles reported in the literature since 2000 (59 articles in total) shows that 75% of them concern therapeutic agents, whereas the remaining 25% concern diagnostic agents (Figure 3). Moreover, the majority of therapeutic agents exert neuroprotective effects (55%), even if anticancer and cardioprotective agents represent two other highly populated classes of therapeutics (16% and 14%, respectively).

## 2. Synthesis of the Benzoylpiperidine Fragment

Analyzing the literature, the preparation of the benzoylpiperidine fragment appears to be a few-step and straightforward synthesis; furthermore, it involves safe and low-cost reagents, thus favoring its use in drug design and development. The presence of the free amino group of the piperidine allows its functionalization thanks to simple nucleophilic substitutions; in addition, if the benzoyl portion of the molecule bears a halogen atom or other functional groups, such as a -SH moiety, it can be subjected to cross-coupling reactions, thus simplifying the obtainment of the desired bioactive molecules.

**Figure 4:**
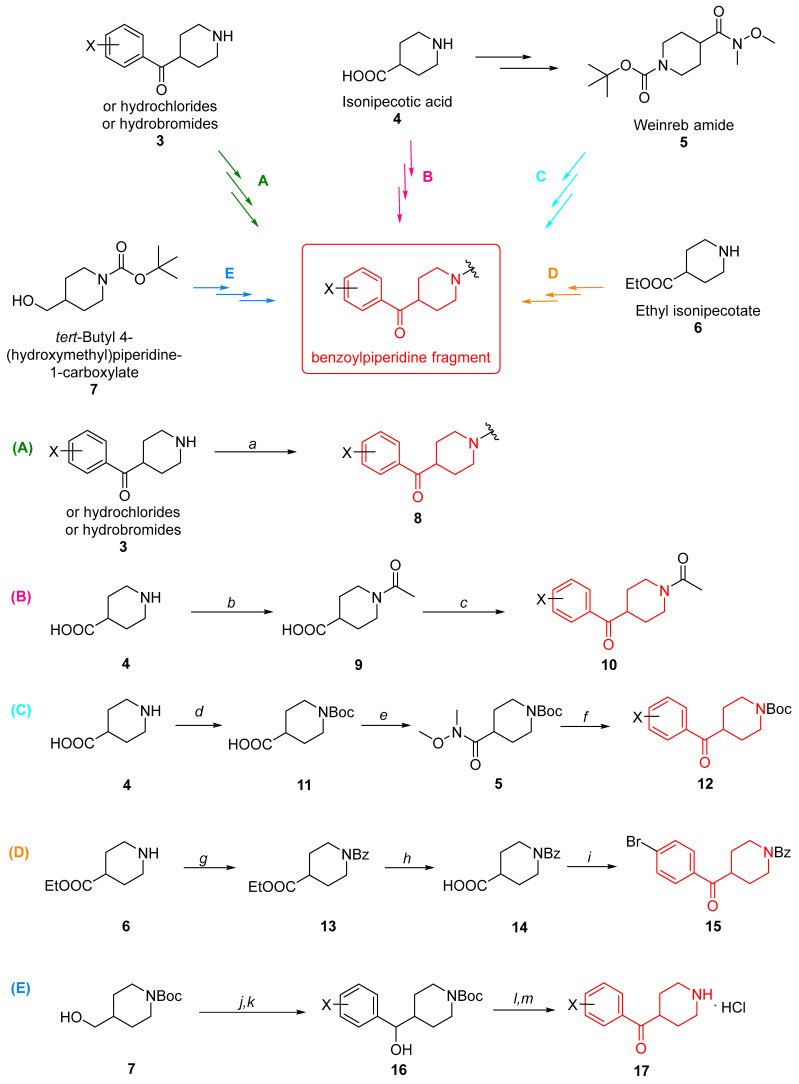
Main commercially available precursors for the synthesis of the benzoylpiperidine fragment (highlighted in red). *Reagents and conditions*: (*a*) nucleophilic substitutions with tosylates, *N*-alkylations with alkylhalides, or amidic condensations with carboxylic acids; (*b*) Ac_2_O, pyridine, 140 °C, 2 h; (*c*) *i.* SOCl_2_, dry 1,2-DCE. Sixty °C, 4 h, *ii.* properly substituted aromatic system, AlCl_3_, dry 1,2-DCE, 90 °C, overnight; (*d*) (Boc)_2_O, 1N NaOH, 1,4-dioxane, acetonitrile, H_2_O; (*e*) *N,O*-dimethylhydroxylamine hydrochloride, HBTU, DIPEA, DMF; (*f*) aromatic organometallic reagent such as a Grignard reagent or organolithium reagent; (*g*) Et_3_N, BzCl, dry DCM, rt, 20 h; (*h*) NaOH, aqueous EtOH (70%), rt, 18 h; (*i*) *i.* (COCl)_2_, dry DCM, rt, 15 h; *ii.* bromobenzene, AlCl_3_, 90 °C, 6.5 h; (*j*) PCC, CH_2_Cl_2_; (*k*) ArMgBr or ArLi, THF; (*l*) Dess-Martin periodinane; (*m*) 4N HCl in 1,4-dioxane.

In most cases, the already-formed benzoylpiperidine fragment is introduced into the structure of bioactive compounds by using commercially available and appropriately substituted amines or their respective hydrochlorides or hydrobromides (**3**, Figure 4), which are subsequently subjected to nucleophilic substitutions with tosylates, *N*-alkylations with alkylhalides, or amidic condensations with carboxylic acids to grow up the molecule on the piperidine nitrogen side (compound **8**, green arrow, sequence of reactions “A”, step *a*, Figure 4) [11,13,14,15,16,17,18,19,20,21,22,23,24,25,26,27,28,29,30,31,32,33,34,35,36,37,38].

In other cases, the benzoylpiperidine fragment is synthesized starting from the commercially available isonipecotic acid (compound **4**, magenta arrow, sequence of reactions “B”, Figure 4). The first step of the synthesis consists of the *N*-acetylation of the free amino group of the piperidine to avoid unwanted side products in the next steps of the synthesis. Indeed, the reaction between the commercial precursor with acetic anhydride and pyridine, refluxed at 140 °C for 2 h, affords the *N*-acetylated intermediate **9** (step *b*, Figure 4). At this point, the treatment of *N*-protected isonipecotic acid with thionyl chloride in anhydrous 1,2-dichloroethane at 60 °C for 4 h gives the acyl chloride intermediate, which is then subjected to Friedel-Crafts acylation with the properly substituted aromatic system in the presence of aluminum trichloride and anhydrous 1,2-dichloroethane as the solvent at 90 °C overnight to form the desired benzoylpiperidine fragment **10** (step *c*, Figure 4). Then, an appropriate sequence of reactions allows the functionalization of the benzoylpiperidine on both the piperidine (after removal of the amide protecting group to obtain the free amine) and the benzoyl moiety [39,40,41,42,43,44].

Weinreb amide **5** (1-*t*Bu-*N*-methoxy-*N*-methylpiperidine-4-carboxamide or *tert*-butyl 4-(methoxy(methyl)carbamoyl)piperidine-1-carboxylate) is another reagent commonly used in the synthesis of the benzoylpiperidine fragment through the Weinreb–Nahm ketone synthesis (light blue arrow, sequence of reactions “C”, Figure 4) [45]. This amide is commercially available, or it can be synthesized starting from isonipecotic acid **4**. Isonipecotic acid **4** is *N*-Boc protected with di-*tert*-butyl dicarbonate and 1N aqueous NaOH in a mixture of 1,4-dioxane and acetonitrile to afford intermediate **11** (step *d*, Figure 4) and then treated with *N,O*-dimethylhydroxylamine hydrochloride in the presence of the coupling reagent hexafluorophosphate benzotriazole tetramethyl uronium, *N*,*N*-diisopropylethylamine as the base, and anhydrous dimethylformamide as the solvent to yield the desired amide **5** (step *e*, Figure 4). The subsequent treatment of Weinreb amide **5** with an aromatic organometallic reagent such as a Grignard reagent or organolithium reagent allows the formation of a new carbon-carbon bond and thus the desired benzoylpiperidine fragment **12** (step *f*, Figure 4) [1,46,47,48,49].

Another strategy adopted to synthesize the benzoylpiperidine fragment is the employment of the commercially available ethyl ester of isonipecotic acid **6** (ethyl isonipecotate, orange arrow, sequence of reactions “D,” Figure 4). The free amino group can be protected with benzoyl chloride in the presence of triethylamine as the base and anhydrous dichloromethane as the solvent at room temperature for 20 h (step *g*, Figure 4). Then, the ethyl ester group of benzamide **13** is hydrolyzed in alkaline conditions for aqueous NaOH in aqueous EtOH (70%) at room temperature for 18 h to obtain free carboxylic acid **14** (step *h*, Figure 3). Here, a sequence of reactions very similar to those previously described allows the assembly of the benzoylpiperidine fragment. The carboxylic acid **14** is first converted into its acyl chloride analogue by using oxalyl chloride in dry DCM at room temperature for 15 h and subsequently reacted with bromobenzene in a Friedel-Crafts reaction in the presence of AlCl_3_ at 90 °C for 6.5 h to afford intermediate **15** possessing the benzoylpiperidine fragment (step *i*, Figure 4). The bromine in the *para* position of the benzoyl moiety allows the easy further functionalization of this portion of the molecule [50,51,52,53,54,55,56,57,58,59,60].

Finally, the benzoylpiperidine fragment can also be obtained starting from the commercially available *tert*-butyl 4-(hydroxymethyl)piperidine-1-carboxylate **7** (blue arrow, sequence of reactions “E”, Figure 4). At first, it is oxidized by pyridinium chlorochromate in dry DCM, and then it is converted into the corresponding secondary alcohol **16** when reacted with arlymagnesium bromide or aryllithium reagents in tetrahydrofuran (steps *j* and *k*, Figure 4). Then, the Dess-Martin oxidation with 1,1,1-tris(acetyloxy)-1,1-dihydro-1,2-benziodoxol-3-(1*H*)-one (Dess-Martin periodinane) followed by the deprotection of the secondary alcohol in acidic conditions for 4N HCl in 1,4-dioxane affords the benzoylpiperidine fragment **17** (steps *l* and *m,* Figure 4) [61,62].

## 3. Benzoylpiperidine-Based Small Molecules as Therapeutics and/or Diagnostics

### 3.1. Cancer

#### 3.1.1. MAGL Inhibitors

Monoacylglycerol lipase is a cytosolic serine hydrolase responsible for 85% of 2-arachidonoylglycerol (2-AG) metabolism in the brain. This enzyme is overexpressed in aggressive cancer cells and primary tumors, where it furnishes a lipolytic source of free fatty acids important to producing oncogenic signaling lipids [63]. For this purpose, the development of MAGL inhibitors is a valid and appealing strategy for tackling cancer. Many irreversible MAGL inhibitors have been developed so far; however, the chronic MAGL blockade causes serious side effects in animal-based studies [64]. Hence, the development of reversible MAGL inhibitors able to partially inhibit MAGL, thus avoiding interference with the endocannabinoid system, has become an increasingly challenging research field.

**Figure 5:**
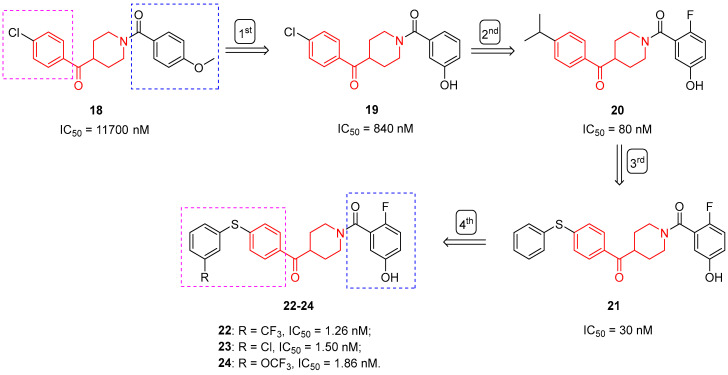
Benzoylpiperidine-based reversible MAGL inhibitors (the benzoylpiperidine fragment is highlighted in red, the benzoyl moiety is in the magenta dashed square and the phenylamidic moiety is in the blue dashed square).

In this context, Tuccinardi et al. discovered a new reversible MAGL inhibitor thanks to the application of computational studies [13]. Compound 18 (Figure 5), characterized by a benzoylpiperidine fragment, proved to inhibit human MAGL with an IC_50_ value of 11.7 µM, acting as a reversible inhibitor, as supported by pre-incubation assays. In addition, Michaelis-Menten analysis suggested competitive behavior for compound **18**. Further pharmacological investigations showed that benzoylpiperidine **18** exerted notable antiproliferative activity on human breast (MDA-MB-231 and MCF-7) and ovarian (COV318 and OVCAR-3) cancer cells compared with noncancerous human mesenchymal stem cells (hMSC), with IC_50_ values ranging from 19.9 to 75.3 µM. These promising results prompted the researchers to further modify and optimize benzoylpiperidine **18** to obtain more potent reversible MAGL inhibitors. Before proceeding with the design, molecular docking studies were performed to better understand the disposition of the ligand inside the active site of the enzyme and thus better direct the chemical modifications to be made to the lead compound **18** [24]. The benzoyl portion of the ligand is located in the wide lipophilic channel of MAGL, establishing lipophilic interactions, whereas the phenylamidic portion is located in the small polar pocket of the enzyme. At first, the researchers improved the interaction of the ligand with the small polar pocket while maintaining the rest of the molecules fixed. So, they replaced the *p*-methoxy group with some exploratory substituents on the different positions of the phenylamidic ring, and among the newly synthesized benzoylpiperidines, compound **19** (Figure 5), bearing a hydroxyl group in *meta* position with respect to the amide, displayed the most potent MAGL inhibition activity, with an IC_50_ value of 0.84 µM [24]. Dilution and preincubation assays confirmed its reversible mode of action, and Michaelis-Menten experiments suggested a competitive behavior, such as the lead compound **18**. Importantly, benzoylpiperidine **19** proved to be selective for MAGL compared with fatty acid amide hydrolase (FAAH), another target enzyme of the endocannabinoid system. Finally, **19** was also tested for its antiproliferative activity on different cancer cell lines, and it displayed a good inhibition of cell viability in ovarian cancer cells OVCAR-3 and COV318 (IC_50_ values of 31.5 and 43.9 µM, respectively), which overexpress MAGL, as demonstrated by Western blot analysis. Computational studies about the binding mode of ligand **19** to the MAGL active site highlighted that the hydroxyl group in the *meta* position established a crucial H-bond with E53 and H272 in the small polar pocket of the enzyme. Taking into account the above-mentioned findings and the fact that the benzoyl portion of compound **19** is located in the wide lipophilic channel of MAGL, the next endeavors of the researchers focused on the structural modification of both the phenolic and the benzoyl moiety of compound **19**, thus leading to the identification of compound **20** (Figure 5) [39]. Benzoylpiperidine **20** possesses an *i*-propyl group in the *para* position of the benzoyl moiety and a fluorine atom in the *para* position of the phenolic group, making it the most potent reversible inhibitor of this series, with an IC_50_ value of 80 nM on the isolated enzyme. It also proved to be selective for MAGL compared with other ECS enzymes and cannabinoid receptors 1 and 2 (IC_50_ > 10 µM). Benzoylpiperidine **20** significantly inhibited the cell growth of different human breast, ovarian, and colorectal cancer cells, with IC_50_ values ranging from 7.9 to 92 µM. The consistent improvement in MAGL inhibition activity from **19** to **20** (of about 10-fold) seemed to be mainly ascribed to the fluorine atom in the *para* position with respect to the phenolic group: its electron withdrawing effect makes the hydroxyl group more acidic, thus reinforcing its H-bonds with E53 and H272 in the small polar pocket of the enzyme. Furthermore, the *i*-propyl group better fitted into the wide lipophilic channel of MAGL and established more hydrophobic interactions within it compared with the chlorine atom. These hypotheses were further confirmed in 2021, when the researchers designed a second-generation class of benzoylpiperidines as reversible MAGL inhibitors [40]. This chemical exploration led to diarylsulfide **21** (Figure 5), which proved to be one of the most promising compounds. Diarylsulfide **21** reversibly inhibited MAGL with an IC_50_ value of 30 nM and proved to be selective for MAGL compared with FAAH, ABHD-6/-12, CB1R, and CB2R (IC_50_ > 10 µM). The potential use of **21** as an anti-cancer drug was supported first by antiproliferative activity assays in a panel of cancer cell lines and secondly by its evaluation in a pancreatic ductal adenocarcinoma (PDAC) preclinical model. In this regard, pharmacological assays on PDAC-3 primary cell cultures showed good antiproliferative activity for compound **21** with an IC_50_ value of 9.28 µM.

With the aim of obtaining even more potent reversible MAGL inhibitors, Bononi et al. further optimized the scaffold of **21** by introducing some exploratory substituents in *the para* or *meta* positions of the distal phenyl ring of the diarylsulfide moiety [41]. The best results in terms of MAGL inhibition activity and selectivity were obtained by diphenylsulfides **22**–**24** bearing a substituent (trifluoromethyl, chlorine, or trifluoromethoxy, respectively, Figure 4) in the *meta* position of the distal phenyl ring. Their IC_50_ values were in the low nanomolar range (1.26, 1.50, and 1.86 nM, respectively). Diarylsulfides **22**–**24** were also endowed with remarkable antiproliferative activity in a panel of nine different cancer cells with IC_50_ values in the range of 0.32–12 µM. The binding mode of this class of diarylsulfide benzoylpiperidine-based MAGL inhibitors highlighted that the *meta*-substituent was able to fully occupy the hydrophobic pocket of the enzyme, interacting with most of its residues; moreover, the phenyl ring reinforced the hydrophobic interactions with the leucine residues of this pocket.

In conclusion, the lead optimization of **18** to obtain compound **22** allowed a notable improvement in MAGL inhibition activity while still maintaining the benzoylpiperidine central fragment fixed.

#### 3.1.2. Tankyrase Inhibitors

Tankyrase (TNKS) proteins are multifunctional poly(ADPribose) polymerases (PARPs) using NAD^+^ as a substrate to generate ADP-ribose polymers on target proteins (PARsylation). This covalent post-translational modification leads to the attachment of one or many ADP-ribose (ADPr) molecules onto the target protein [65]. TNKS1 and TNKS2 isoforms share identical functions and comparable structures, including the ankyrin (ANK) repeat domain, the SAM (sterile alpha molecule) domain, and the catalytic PARP domain [65]. It was found out that TNKS is a feasible target to inhibit the Wnt/β-catenin signal transduction that is improperly activated in many cancers [66,67]. It is not yet clear the mechanism of action, but TNKS seems to favor the ubiquitination of axin through its direct PARsylation [66]. Therefore, TNKS inhibitors may be developed as novel anti-cancer drugs mining the Wnt signaling pathway.

**Figure 6:**
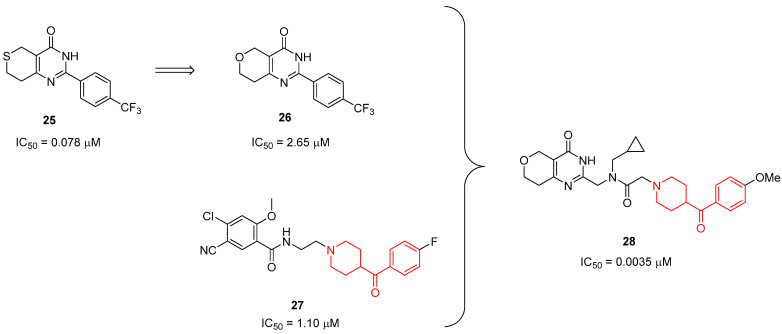
Design and structure of Tankyrases inhibitors as potential anti-cancer agents. IC_50_ values were evaluated in the HEK293 SuperTopFlash (STF) reporter gene assay. The benzoylpiperidine fragment is highlighted in red.

Within this context, Shultz and colleagues, by using a combination of structure-based design and lipophilic efficiency (LipE)-based structure efficiency relationships, optimized the previously developed TNKS inhibitor **25** aiming at identifying a more stable and efficient derivative (Figure 6) [32]. The first structural modification led to dihydropyran **26** (Figure 6), which exhibited a better stability and lipophilicity profile, but it was 33-fold less potent than **25** (IC_50_ values of 2.65 and 0.078 µM, respectively, in human embryonic kidney HEK293 cells SuperTopFlash reporter gene assay). Hence, this core was further combined with the chemical features of compound **27**, a screening hit discovered in the same study and characterized by a benzoylpiperidine moiety (Figure 6). A new series of 10 benzoylpiperidine-based TNKS inhibitors was synthesized, starting with commercially available *p*-substituted benzoylpiperidines. The most potent and selective TNKS inhibitor of this series was found to be compound **28** (Figure 6), possessing an increased LipE of 7.0 (the LipE for **25** and **26** was 4.2 and 4.0, respectively) and a 5000-fold selectivity for TNKS1 and TNKS2 versus PARPs 1 and 2. Pharmacokinetic studies in mice revealed that **28** is an orally active antagonist of the Wnt pathway with favorable pharmacokinetic properties compared with the parent compound **25**. Taken together, these results paved, the way for the development of novel TNKS inhibitors based on this chemical structure to treat Wnt-dependent cancers.

#### 3.1.3. Complex I Inhibitors

NADH-ubiquinone oxidoreductase, also known as Complex I, is the first oxidative phosphorylation (OXPHOS) enzyme that oxidizes NADH generated in the tricarboxylic acid (TCA) cycle and in the fatty acid β-oxidation to regenerate NAD^+^ for the mitochondrial matrix. Moreover, it uses the two electrons to reduce ubiquinone to ubiquinol [68,69]. Despite the fact that cancer cells mainly rely on glycolysis to produce energy (the Warburg effect), some types of cancer still rely on OXPHOS [70]. Hence, Complex I inhibitors interfering with OXPHOS could be a valuable approach to counteracting specific cancers.

**Figure 7:**
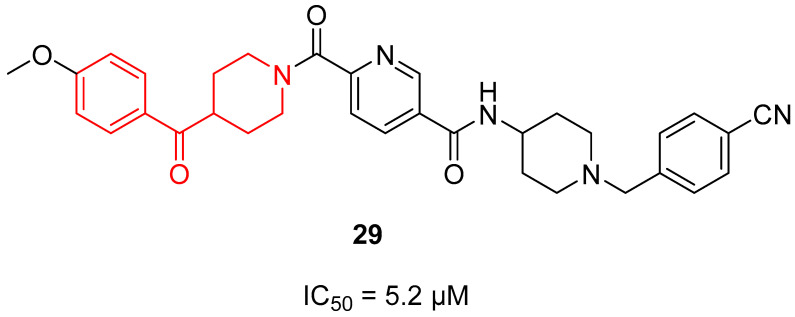
Structure of **29** Complex I inhibitor (benzoylpiperidine highlighted in red).

In 2013, compound **29** (Figure 7) was identified as a Complex I inhibitor able to activate AMPK in vitro, thus ameliorating type 2 diabetes and its complications (see Section 3.4.2 AMPK activators) [22]. Based on these findings, some years later, Huang and coworkers decided to investigate the anti-cancer activity of **29** on the human liver cancer cell line HepG2 and optimize its chemical scaffold [71]. While **29** is characterized by a benzoylpiperidine fragment in its structure (as highlighted in red in Figure 7), the newly 20 synthesized **29** derivatives did not possess this chemical feature. However, focusing on **29**, it exerted a notable antiproliferative activity on HepG2, with an IC_50_ value of 5.2 µM, displaying a dose-dependent and moderate inhibitory activity of Complex I.

### 3.2. Neuropsychiatric and Neurodegenerative Diseases

#### 3.2.1. Serotoninergic and Dopaminergic Receptor Ligands

Nowadays, it is well known that the neurotransmitter serotonin (5-hydroxytriptamine, 5-HT) plays a central role in the occurrence of mental diseases such as depression, anxiety, schizophrenia, eating disorders, obsessive-compulsive disorder (OCD), migraine, and panic disorder [72,73,74,75,76,77]. Therefore, modulating the serotoninergic tone is a commonly used strategy for treating mental disorders. The 5-HT receptors are divided into seven families (5-HT_1–7_), and some of these families are further classified into different subtypes (A, B, C, and so on) [78]. For example, the 5-HT_1A_ receptor is implicated in neuropsychiatric disorders such as anxiety and depression [79], whereas the 5-HT_2A_ receptor is correlated with mental illness and cardiovascular diseases [80]. As mentioned before, two representative examples of 5-HT_2A_ ligands are compounds **1** and **2**, both characterized by a benzoylpiperidine fragment (Figure 1). Ketanserin **1** is mainly used in clinics as an antihypertensive agent, while altanserin **2** is usually labeled with the isotope fluorine-18 to form the respective radioligand that is employed in positron emission tomography (PET) studies of the brain.

Together with the serotoninergic system, the dopaminergic one plays a central role in schizophrenia, as first hypothesized by Van Rossum in 1967; that is, dopamine neurotransmission is increased in this illness [81]. The dopaminergic receptors are classified as D_1_- and D_2_-like. The D_2_-like family is constituted by D_2_, D_3_, and D_4_ receptors, and it seems to be directly involved in the onset of schizophrenia. Antipsychotic drugs are divided into two classes on the basis of the Meltzer’s ratio, which is the p*K*_i_ (5-HT_2A_/D_2_) ratio: (*a*) typical (classical, Meltzer’s ratio < 1.09); and (*b*) atypical (non-classical, Meltzer’s ratio > 1.12) antipsychotics. The first ones are characterized by different side effects such as prolactin release and extrapyramidal symptoms (EPS), and they are also ineffective against negative symptoms (social withdrawal, catatonia, and affective flattening of the personality); on the contrary, the second ones do not provoke EPS and can treat both negative and positive (hallucinations, paranoia, and disorganized behavior) symptoms. This difference between the two classes of antipsychotics depends on the fact that atypical antipsychotics block not only dopamine receptors but also serotonin receptors. Therefore, many endeavors in this research field have also focused on the identification of dual 5-HT_2A_/D_2_ ligands with a Meltzer’s ratio greater than 1.12.

In the development of new antipsychotic drugs targeting the serotoninergic and dopaminergic systems, the benzoylpiperidine frame is very recurrent for the reasons already mentioned in Section 1 [9,10,11,12].

**Figure 8:**
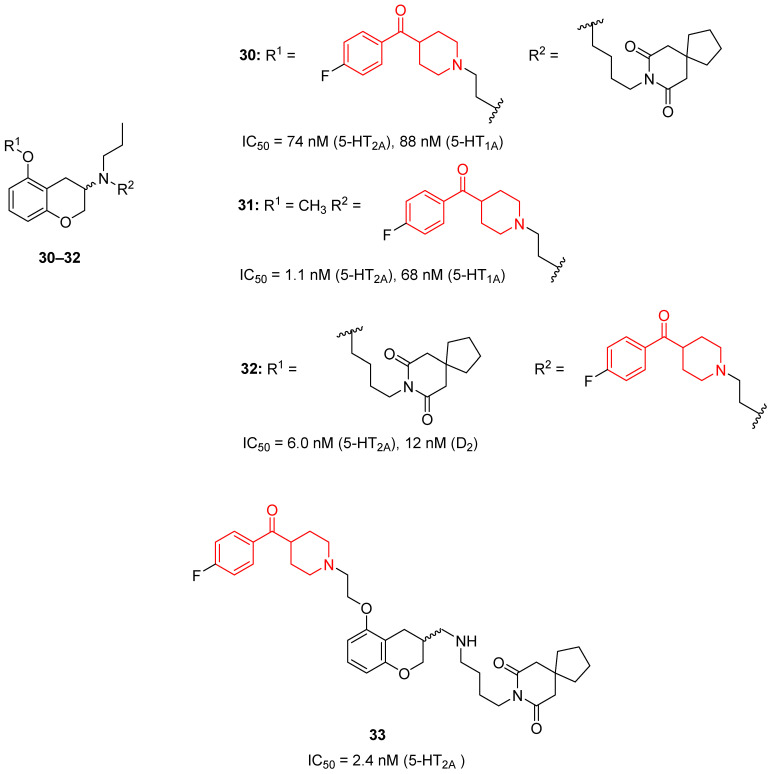
Structure of compounds **30**–**33** developed by Guillaumet et al. [33] possessing a benzoylpiperidine fragment (in red).

In 2000, the research group of Guillaumet aimed at developing new molecules with a mixed affinity for 5-HT_1A_ and 5-HT_2A_ receptors by modifying the structures of previously developed compounds endowed with a high affinity for 5-HT_1A_ receptors [33]. Among the 10 synthesized compounds, four of them possess a 4-(*p*-fluorobenzoyl)piperidine moiety (compounds **30**–**33**, Figure 8).

As a result of the biological evaluation of these derivatives, they identified compounds **31** and **33** as potent and selective 5-HT_2A_ ligands with IC_50_ values of 1.1 and 2.4 nM, respectively. Compound **31** also demonstrated to bind to high-affinity 5-HT_1A_ receptors with an IC_50_ value of 68 nM. On the other hand, compound **32** acted as a mixed ligand for 5-HT_2A_ and D_2_ receptors with IC_50_ values of 6.0 and 12 nM, respectively. Finally, compound **30** proved to be a mixed 5-HT_1A_/5-HT_2A_ ligand with similar activities for both receptors, showing IC_50_ values of 88 and 74 nM, respectively.

In the same year, the research group of Prof. Masaguer and Prof. Loza synthesized and pharmacologically evaluated 2-aminomethyl-1,2,3,9-tetrahydro-4*H*-carbazole-4-ones as potential atypical antipsychotics [11]. Compound **34** (Table 1) turned out to be one of the most interesting compounds in this series, and it was characterized by a benzoylpiperidine frame in its chemical structure. Compound **34** possessed a notable affinity for 5-HT_2A_ receptors (p*K*_i_ = 8.04) and a lower affinity for D_2_ receptors (p*K*_i_ = 6.25). Its p*K*_i_ (5-HT_2A_/D_2_) ratio of 1.29 suggested an atypical antipsychotic profile according to Meltzer’s classification. In addition, compound **34** was also demonstrated to be selective for 5-HT_2A_ receptors compared with 5-HT_2B_ and 5-HT_2C_ receptors by about 100- and 60-fold, respectively.

The structures of compound **34** and of the most representative benzoylpiperidines developed by the research group of Prof. Masaguer and Prof. Loza as antipsychotic agents and discussed in the following paragraphs are summarized in Table 1.

Shortly after, the same research group synthesized 6-aminomethyl-4,5,6,7-tetrahydrobenzo[b]furan-4-ones, hoping to afford antipsychotic agents with reduced EPS [34]. One of the most active compounds in this series was compound **35** (Table 1), characterized by a 4-(*p*-fluorobenzoyl)piperidine moiety. Derivative **35** was subjected to biological assays in order to evaluate its ability to bind serotoninergic (5-HT_2A_, 5-HT_2C_, and 5-HT_2B_) and dopaminergic (D_1_, D_2_, and D_4_) receptors. Compound **35** showed a notable affinity for 5-HT_2A_, D_1_, D_2_, and D_4_ receptors, a moderate selectivity for 5-HT_2B_, and a scarce affinity for 5-HT_2C_ receptors. Its 1.03 p*K*_i_ 5-HT_2A_/D_2_ ratio is in the range of that of typical or classical antipsychotics, and this justified the fact that **35** induces catalepsy less than the reference compound haloperidol, thus suggesting its scarce ability to provoke acute EPS as a side effect. All these results supported the possibility of using compound **35** as a neuroleptic drug.

**Figure 9:**
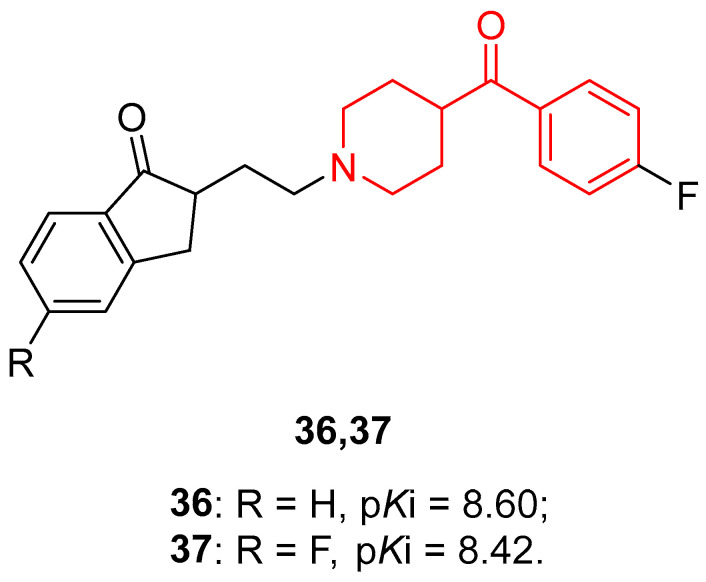
Structure of the 5-HT_2A_ ligands **36**,**37** possessing a benzoylpiperidine fragment (in red).

Other classes of antipsychotic drugs were developed by these researchers, and in 2002 they conducted an in-depth SAR analysis of 52 cycloalkanones, many of them linked to a benzoylpiperidine fragment [82]. The most interesting benzoylpiperidine-based cycloalkanones of this series were compounds **36** and **37** (Figure 9), previously synthesized in 1994 [83], which proved to be promising 5-HT_2A_ ligands with p*K*_i_ 5-HT_2A_ of 8.60 and 8.42, respectively.

These two molecules were also selective for 5-HT_2A_ receptors over 5-HT_2B_ receptors, with a *K*_B_ (5HT_2A_/5HT_2B_) ratio of 49 and 100, but also over 5-HT_2C_ receptors, with a *K*_i_ (5HT_2A_/5HT_2C_) ratio of 66 and 219, respectively.

Further investigations into this class of compounds led to the optimization of the previously developed compound **38** (Table 1), thus leading to the synthesis of derivative **39** (Table 1) [35]. The differences in affinity for 5-HT_2A_, 5-HT_2C_, and D_2_ receptors for the corresponding enantiomers (*R*)/(*S*) were irrelevant. Despite this, benzoylpiperidine-aminomethyltetralone (−)-**39** showed a p*K*_i_ 5-HT_2A_/D_2_ ratio of 1.37, suggesting an atypical antipsychotic profile, according to Meltzer’s ratio, with a notable affinity for 5-HT_2A_ (p*K*_i_ = 8.25) and selectivity over D_2_ receptors (p*K*_i_ = 6.00) of about 177-fold.

The replacement of the benzene ring of the tetralone core of compound **38** by an aromatic heterocyclic ring, such as pyrimidine or pyridazine, afforded 7-((4-(4-fluorobenzoyl)piperidin-1-yl)methyl)-7,8-dihydroquinazolin-5(6*H*)-one **40** and 7-((4-(4-fluorobenzoyl)piperidin-1-yl)methyl)-3-methyl-7,8-dihydrocinnolin-5(6*H*)-one **41**, respectively (Table 1) [36]. The Tetrahydro-quinazolinone derivative **40** proved to be the most potent ligand for both 5-HT_2A_ and D_2_ receptors (*K*_i_ of 32 and 160 nM, respectively), endowed with an appreciable selectivity for 5-HT_2A_ over 5-HT_2C_ receptors of about 16-fold. In addition, Meltzer’s ratio of 1.10 classified compound **40** at the limit of an atypical antipsychotic profile. On the other hand, tetrahydro-cinnolinone derivative **41** did not show affinity for D_2_ receptors (*K*_i_ > 10 µM), whereas it exhibited the highest affinity for 5-HT_2C_ receptors (*K*_i_ = 36 nM) with a remarkable selectivity over 5-HT_2A_ (265-fold) and D_2_ (280-fold) receptors.

Unfortunately, the further introduction of exploratory substituents in the 2-position of the pyrimidine ring of compound **40**, such as in the 4-fluorobenzoylpiperidine derivative **42** (Table 1), did not lead to the development of potent D_2_/5-HT_2A_ ligands [14].

**Figure 10:**
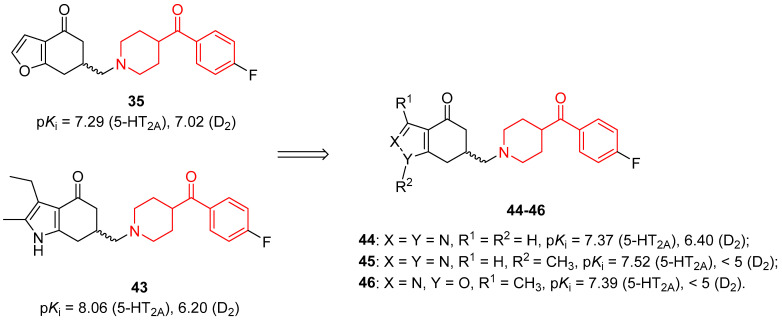
Optimization of 5-HT_2A_ ligands **35** and **43** and structure of compounds **44**–**46**. Benzoylpiperidine fragment is highlighted in red.

Continuing with the development of new potential atypical antipsychotics, the furan or the pyrazole rings of compounds **35** [34] and **43** (Figure 10) [84], respectively, were substituted with isoxazole and pyrazole to obtain tetrahydro-benzisoxazol-4-one and tetrahydro-indazol-4-one derivatives [37]. Some of these conformationally constrained aminobutyrophenones also contained benzoylpiperidine fragments, such as compounds **44**–**46** (Figure 10).

Compounds **45** and **46** showed a very low affinity for D_2_ receptors (p*K*_i_ < 5); thus, they could not be considered as promising antipsychotics, even if tetrahydro-benzisoxazol-4-one **46** demonstrated a good selectivity for 5-HT_2A_ (p*K*_i_ = 7.39) over 5-HT_2C_ (p*K*_i_ < 5) and D_2_ (p*K*_i_ < 5) receptors. Tetrahydro-indazol-4-one **44** exhibited a good affinity for 5-HT_2A_ (p*K*_i_ = 7.37) and a modest affinity for D_2_ (p*K*_i_ = 6.40) receptors, and it was inactive on 5-HT_2C_ receptors (p*K*_i_ < 5); however, the most active compound of this series was a tetrahydro-indazolone bearing a 6-fluorobenzisoxazolylpiperidine instead of the 4-fluorobenzoylpiperidine fragment.

In 2008, the further optimization of compound **35** [34] by introducing different substituents in the 2- or 3-position of the furan ring led to aminoalkylbenzofuran-4-one **47**–**49** (Table 1) [38]. The three compounds, characterized by the *p*-fluorobenzoyl moiety, remarkably bound 5-HT_2A_ receptors (p*K*_i_ ranging from 7.59 to 7.76), and they did not show affinity for D_2_ and 5-HT_2C_ receptors (p*K*_i_ < 5). These results were supported by molecular docking analysis: benzoylpiperidines **47**–**49** established a H-bond with serine at position 3.36 of the 5-HT_2A_ receptor and no H-bond with cysteine at position 3.36 of the D_2_ receptor.

**Figure 11:**
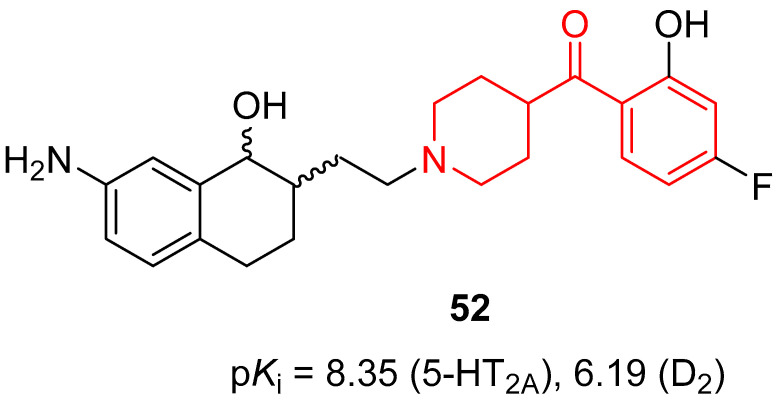
Structure of the 5-HT_2A_ ligand **52** possessing a benzoylpiperidine fragment (in red).

The research group of Prof. Masaguer and Prof. Loza continued to develop new atypical antipsychotics with a dual antagonism versus 5-HT_2A_ and D_2_ receptors, and in 2011, they synthesized and evaluated heterocyclic bioisosteric butyrophenone analogues of the previously described 5-HT_2A_/D_2_ antagonists [16]. Among the 15 newly developed compounds, some of them possessed the benzoylpiperidine fragment (compounds **50**, **51**, Table 1); however, tetrahydrobenzoxazol-4-one **50** and benzothiazol-7-one **51** showed a moderate affinity for 5-HT_2A_ receptors (p*K*_i_ values of 6.90 and 6.55, respectively) and a very scarce affinity for D_2_ receptors, thus not being useful as antipsychotics for the treatment of schizophrenia. On the contrary, another structural modification of compound **38** [82] led to the identification of new potential dual 5-HT_2A_/D_2_ ligands [24]. One of them was a 1-tetralol derivative **52** bearing a 4-(4-fluoro-2-hydroxybenzoyl)piperidinyl fragment in its structure (Figure 11).

1-Tetralol derivative **52** displayed a notable affinity for 5-HT_2A_ receptors (p*K*_i_ of 8.35), higher than that of the reference compounds haloperidol and clozapine (p*K*_i_ of 6.78 and 8.04, respectively). Moreover, ligand **52** showed a moderate affinity for D_2_ receptors (p*K*_i_ of 6.19) with a Meltzer’s ratio of 1.35, indicating an atypical profile. Functional assays (p*K*_B_) on human D_2_ and 5-HT_2A_ receptors highlighted its competitive neutral antagonism profile at both receptors (p*K*_B_ D_2_ of 6.32 and p*K*_B_ 5-HT_2A_ of 7.82).

**Figure 12:**
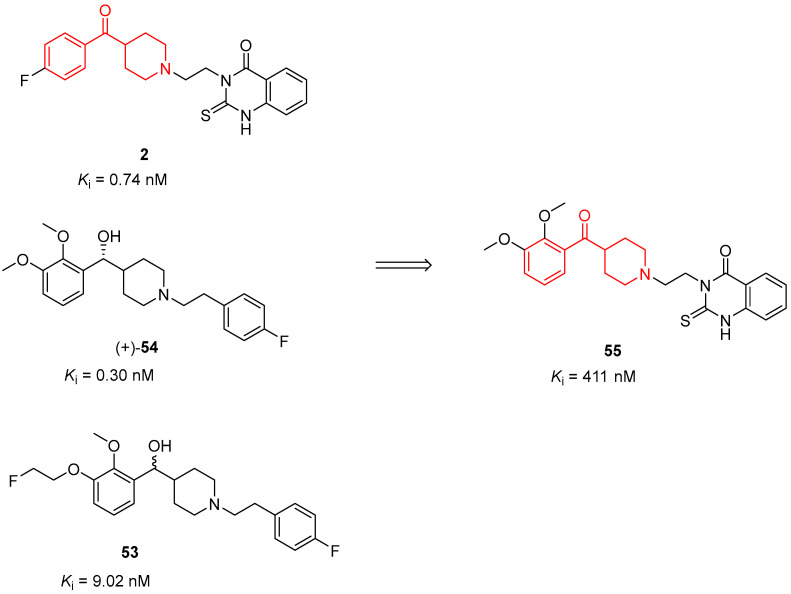
Structure of the 5-HT_2A_ selective antagonists **2**, (+)-**54**, **53**, and the developed ligand **55** possessing a benzoylpiperidine fragment (in red).

In the early 2010s, taking into account that altanserin (**2**), MH.MZ (**53**) and (+)-MDL-100907 (Volinanserin, **54**) (Figure 12) are structurally similar to benzoylpiperidine compounds, Kramer et al. decided to combine the structural elements of these well-known 5-HT_2A_ selective antagonists to better evaluate the binding mode of these compounds and to improve the binding properties of the newly developed derivatives [15]. Among the synthesized compounds, **55** was characterized by a benzoylpiperidine fragment (Figure 12).

Unfortunately, the chemical modifications of **55** caused an important decrease in the affinity to bind 5-HT_2A_ receptors (*K*_i_ value of 411 nM) compared with the reference compounds **2**, (+)-**54** and **53** (*K*_i_ of 0.74, 0.30, and 9.02 nM, respectively).

**Figure 13:**
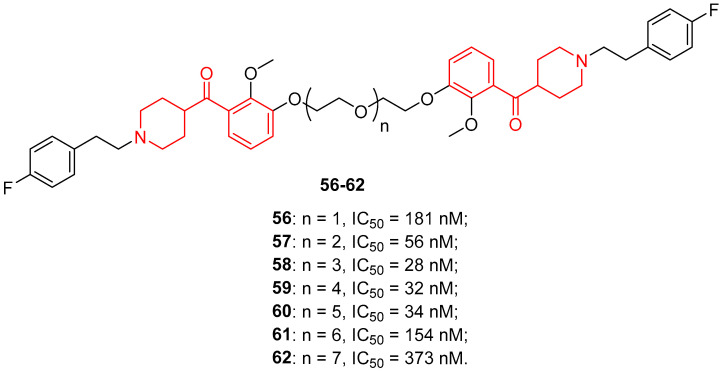
Structure of the 5-HT_2A_ dimeric ligands **56**–**62** possessing a benzoylpiperidine fragment (in red).

In the first decade of the 2000s, evidence suggested that some 5-HT receptors exist as dimers and maybe as oligomers [85,86]. Therefore, the research group at Gilbertson decided to improve the affinity and selectivity of ligands for a given receptor by developing multivalent ligands. In particular, they synthesized dimeric derivatives of the 5-HT_2A_ antagonist (+)-**54** (homodimers **56**–**62**, Figure 13) [46]. They preferred to replace the alcoholic moiety of (+)-**54** with a ketone group to avoid complications during purification and biological testing due to the chiral center of the secondary alcohol of (+)-**54**.

Biological assays highlighted that the antagonist potency over 5-HT_2A_ receptors improved by increasing the length of the linker (from an IC_50_ value of 181 nM of 6 atom-linker **56** to an IC_50_ value of 34 nM of 18 atom-linker **60**), whereas homodimers with 21 and 24 atoms as linkers (compounds **61** and **62**) drastically reduced the potency against the targeted receptors (IC_50_ values of 154 and 373 nM, respectively). However, the best antagonist potency was still obtained with (+)-**54** (IC_50_ value of 3.3 nM).

In 2018, the previously developed bivalent 5-HT_2A_ receptor antagonists **56**–**62** were also biologically profiled thanks to the quantification of extracellular regulated kinases 1/2 (ERK_1/2_) phosphorylation, which is a downstream signaling outcome of 5-HT_2A_ receptor activation [47]. The designed bivalent ligands maintained potency and efficacy on the 5HT_2A_ receptor both in vitro and in vivo when compared with the parent compound (+)-**54**. Dimeric ligands **56**–**62** showed a nanomolar affinity for the 5-HT_2A_ receptor and proved to be selective versus the 5-HT_2B_ and 5-HT_2C_ receptors. Moreover, they displayed nanomolar potency in inhibiting 5-HT-evoked ERK_1/2_ activation, and compounds characterized by linkers of 8–18 atoms were the best to suppress ERK_1/2_ activation. Finally, homobivalent ligand **58** was able to suppress cocaine-evoked behavior in vivo similarly to the parent compound (+)-**54**.

5-HT_7_ is another serotonine receptor involved in cognitive disorders; it is a G_αs_-protein-coupled receptor that plays a main role in the regulation of human CNS functions. Noteworthy, the pathologies determined by 5-HT_7_ receptor disfunctions seem to be strictly correlated with 5-HT_2A_ receptor disorders [87,88,89]. For this reason, the identification of dual ligands of the 5-HT_7_ receptor and the 5-HT_2A_ receptor is considered a valuable approach to treating CNS and psychiatric disorders.

**Figure 14:**
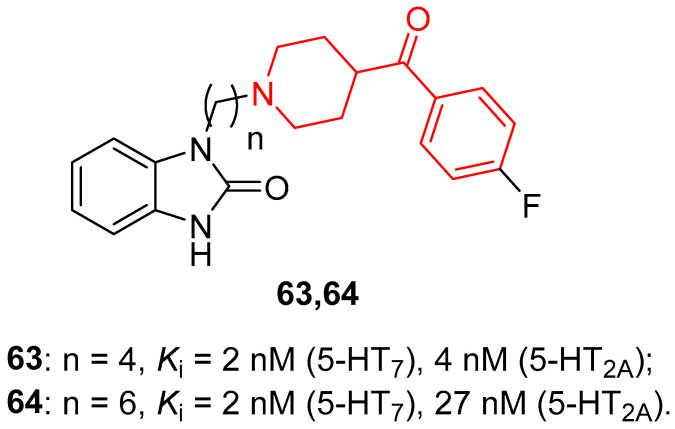
Structure of the dual 5-HT_7_^/^5-HT_2A_ ligands **63**,**64** possessing a benzoylpiperidine fragment (in red).

For this purpose, Deau and colleagues designed and synthesized dual 5-HT_7_/5-HT_2A_ receptor antagonists, and the most promising compounds of this series possessing a fluorine atom in their structure were radiolabeled with [^18^F]fluoride to evaluate their capacity to cross the blood-brain barrier (BBB) by [^18^F]-PET imaging in primate brains [50]. Among the many synthesized compounds, the most representative of them are compounds **63** and **64** (Figure 14), characterized by an alkyl chain of 4 (*n*-butyl) or 6 (*n*-hexyl) carbon atoms (for compounds **63** and **64**, respectively).

In vitro evaluation of these compounds highlighted that **63** and **64** were potent ligands of 5-HT_7_ receptors, both displaying *K*_i_ values of 2 nM and endowed with considerable selectivity compared with 5-HT_6_ and 5-HT_1A_ receptors. Moreover, they showed a very high affinity for 5-HT_2A_ receptors, with *K*_i_ values of 4 nM for **63** and 27 nM for **64**. Results of functional assays confirmed the behavior of compound **63** as a partial antagonist of the 5-HT_7_ receptor and of compound **64** as a full antagonist of the 5-HT_7_ receptor. Both benzoylpiperidine derivatives **63** and **64** proved to be potent and full antagonists of the 5-HT_2A_ receptor. To investigate the ability of these compounds to cross the BBB and their metabolic stability, PET scan imaging analyses on primate brains were carried out, and [^18^F]-**63** and [^18^F]-**64** were radiosynthesized.

Primate brain PET imaging assays evidenced a moderate uptake of radiolabeled molecules [^18^F]-**63** and [^18^F]-**64** with a significant accumulation in the brain stem, thalamus, hippocampus, and anterior cingulate, thus suggesting a considerable presence of 5-HT_7_ receptor and/or 5-HT_2A_ receptor in those brain regions. In addition, both radiotracers displayed good metabolic stability in plasma. Taken together, all the results supported the further development of compounds **63** and **64** as feasible dual 5-HT_7_/5-HT_2A_ receptor antagonists for treating CNS disorders.

Psychiatric disorders, such as schizophrenia, depression, and anxiety, may also be counteracted by targeting metabotropic glutamate (mGlu) receptors, considering their regulatory functions in glutamatergic transmissions. Especially mGlu_2/3_ receptor agonists demonstrated to be effective in the treatment of schizophrenia and anxiety disorders in clinical studies, thus proving the reliability of using mGlu receptor ligands to cure psychiatric disorders [90]. Noteworthy recent findings evidenced the existence of a heteromeric 5-HT_2A_/mGlu_2_ complex [91,92,93], and therefore, the design of a heterobivalent ligand, bearing two receptor-selective head groups separated by a chemical spacer of suitable length and flexibility, represents an appealing approach to selectively target a heteromeric receptor complex.

**Figure 15:**
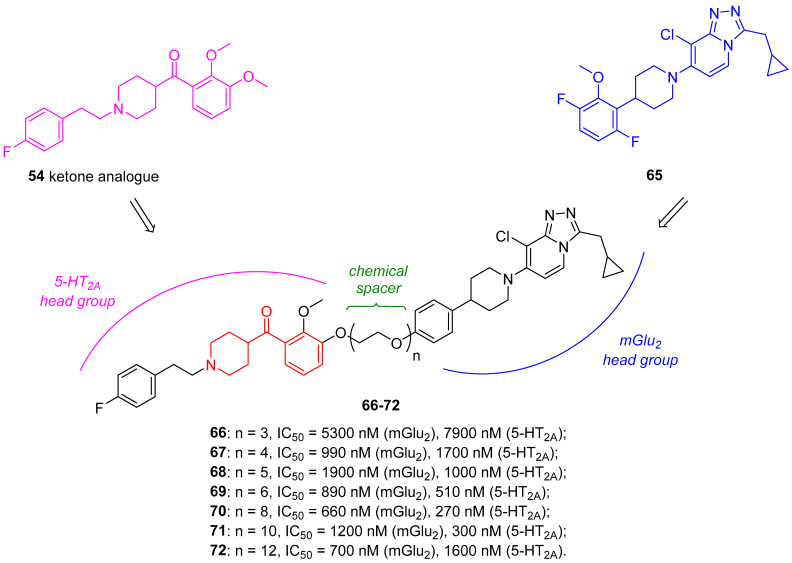
Design and structure of 5-HT_2A_/mGlu_2_ dimeric ligands **66**–**72** possessing a benzoylpiperidine fragment (in red).

To this end, Poulie and coworkers developed heterobivalent ligands for the putative 5-HT_2A_/mGlu_2_ receptor complex characterized by a 5-HT_2A_ head group connected to a mGlu_2_ head group by a proper spacer [49]. As the 5-HT_2A_ head group, they chose the ketone analogue of the potent and selective 5-HT_2A_ antagonist **54**, whereas the mGlu_2_ head group was represented by the low-nanomolar selective and allosteric mGlu_2_ agonist **65** (Figure 15). As the chemical linker between the two head groups, they selected polyethylene glycol (PEG) chains of different lengths to balance the lipophilicity of the molecules (Figure 15).

The pharmacological characterization of these dimeric ligands highlighted that heterobivalent ligands **66**–**72** inhibited 5-HT-induced responses in 5-HT_2A_/mGlu_2_ cells and both 5-HT- and Glu-induced responses in HEK293 cells coexpressing the receptors 5-HT_2A_/mGlu_2_/Gqo5. Nevertheless, the mechanism of interaction between the heterodimeric ligands and the putative 5-HT_2A_/mGlu_2_ receptor complex still remains unclear.

#### 3.2.2. GlyT1 Inhibitors

The pathophysiology of schizophrenia has been demonstrated to also be associated with *N*-methyl-D-aspartate (NMDA) receptor hypofunction. Indeed, the activation of the NMDA receptor and the restoration of the function of glutamatergic neurons represent a valuable approach to treating schizophrenia [94,95]. In spite of that, the NMDA receptor is broadly distributed in the brain, and its direct activation could provoke severe neurotoxic adverse effects and seizures [96]. A possible way to bypass this problem may be by targeting the glycine transporters (GlyTs). Glycine is an obligatory co-agonist of the NMDA receptor, and the activation of the NMDA receptor is promoted by augmenting glycine concentrations in the synaptic cleft [97]. GlyTs are responsible for the uptake of glycine into neurons or surrounding glia. There are two types of GlyTs in the brain: GlyT1, localized to the NMDA receptor area, and GlyT2, which is distributed around the glycinergic neurons [98]. Hence, selective GlyT1 inhibitors increase glycine levels in the glutamatergic synapse and boost NMDA receptor activity, thus being useful to treat schizophrenia.

**Figure 16:**
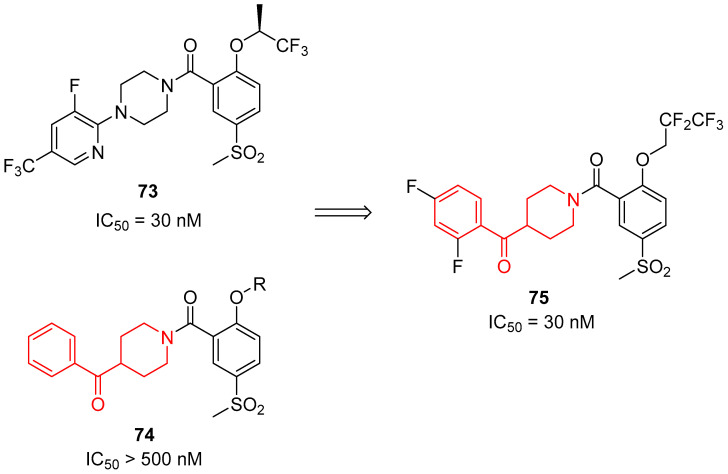
Structure of **73**, general structure **74**, and GlyT1 inhibitor **75** possessing a substituted benzoylpiperidine fragment (in red).

In 2015, Liu and colleagues decided to develop new selective GlyT1 inhibitors [1], starting from the structures of inhibitors already present in the literature: compound **73** (Figure 16) [99] and compounds with a general structure of **74** (Figure 16) [100]. Thus, they designed benzoylpiperidine derivatives (Figure 16), taking into consideration that piperidine is a potential bioisostere of piperazine and that by introducing other heteroatom-containing moieties (in this case, a carbonyl group) in the structure, it is possible to compensate for the reduction in energy binding due to the loss of the second nitrogen atom of piperazine, which is an important site for the formation of hydrogen bonds. Additionally, the benzoylpiperidine fragment is considered a privileged structure used in the discovery of antipsychotic agents [10,11,12].

All the synthesized derivatives were evaluated in vitro for their capacity to inhibit GlyT1, and the most promising results were obtained for benzoylpiperidine **75,** characterized by a 2,2,3,3,3-pentafluoropropoxy substituent (-OCH_2_CF_2_CF_3_) in 2-position and a methylsulfonyl group (-SO_2_CH_3_) in 5-position on the amidic portion and by a 2,4-difluorobenzoyl moiety. Benzoylpiperidine **75** possessed an IC_50_ value of 30 nM and a notable selectivity versus GlyT2 and dopaminergic receptors. Moreover, benzoylpiperidine **75** considerably improved the cognitive deficit in the chronic phencyclidine (PCP)-induced schizophrenia-like animal models when injected at a 40 mg/kg dosage. This very high dosage was mainly due to its scarce ability to cross the BBB, as indicated by the evaluation of its blood/plasma (B/P) ratio of 0.03 and its limited pharmacokinetic profile. These results prompted the researchers to substitute the benzoylpiperidine moiety with another privileged scaffold for the development of antipsychotics, the 3-(piperidin-4-yl)benzo[*d*]isoxazole, but unfortunately, this preliminary optimization did not ameliorate the pharmacokinetic parameters.

#### 3.2.3. σ_1_ Receptor Inhibitors

σ_1_ receptor is a chaperone protein involved in many functions regarding the central nervous system (CNS), where it regulates: neurotransmitter release, modulation of neurotransmitter receptor functions, learning, memory, and posture control [101,102]. Moreover, σ_1_ receptors are overexpressed in different types of cancer, such as breast cancer, colon carcinoma, renal carcinoma, prostate cancer, glioblastoma, neuroblastoma, melanoma, sarcoma, brain tumors, and non-small-cell lung carcinoma [103,104,105,106]. For these reasons, σ_1_ receptor inhibitors are considered feasible therapeutic agents to counteract cancer, neuropsychiatric, and neurodegenerative diseases.

**Figure 17:**
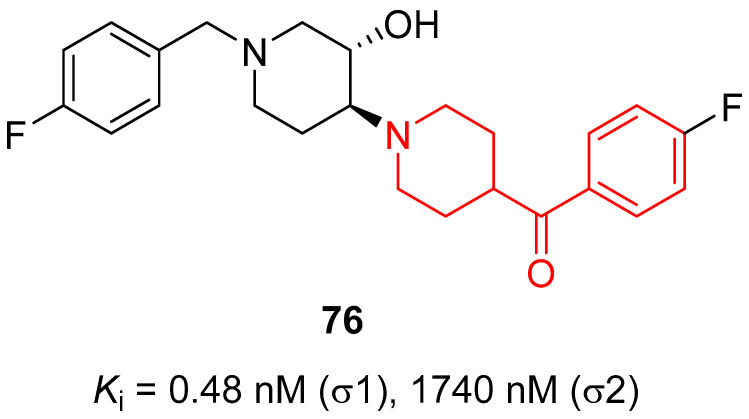
Structure of the most active σ_1_ inhibitor **76** developed by Wang et al. [18], possessing a benzoylpiperidine fragment (in red).

In 2011, Wang and collaborators identified new σ_1_ receptor inhibitors characterized by a benzoylpiperidine fragment [18]. Among this class, racemic compound **76** (Figure 17) demonstrated itself to be the most potent and selective compound of this series. This derivative bears a fluorine atom in the *para* position of the benzoyl moiety, whereas the nitrogen atom of the piperidine core was linked to a 4-fluorobenzyl-3-hydroxy-piperidinyl group. The presence of two fluorine atoms in the structure of **76** made this compound easily labeled with fluorine-18, and therefore it could be developed not only as a therapeutic agent but also as a PET tracer for σ_1_ receptor imaging in vivo. Racemic compound **76** showed the highest affinity for σ_1_ receptor, with a *K*_i_ value of 0.48 nM, and the highest selectivity for σ_1_ receptor compared with σ_2_ receptor (3627-fold) and for σ_1_ receptor versus the vesicular acetylcholine (ACh) transporter (2833-fold). In addition, compound **76** displayed a very low affinity for D_1_, D_2_, and 5HT_1A_ receptors, thus confirming its selectivity for the σ_1_ receptor and the possibility to label compound **76** with ^11^C or ^18^F without affecting the receptor binding process or the imaging signals for PET studies.

**Figure 18:**
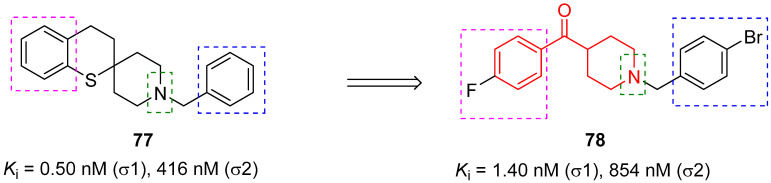
Design and structure of σ_1_ inhibitor **78** developed by Prof. Efange’s research group possessing a benzoylpiperidine fragment (in red). The common fundamental chemical features of compounds **77** and **78** are highlighted in magenta, blue and green dashed squares.

In 2016, the research group of Professor Efange sought to elucidate the role of spirofusion of spipethiane (compound **77**, Figure 18) in the biological activity of the σ_1_ receptor [19]. Spipethiane (**77**) is a very potent and selective ligand of the σ1 receptor, with *K*_i_ values of 0.50 and 416 nM for the σ1 and σ_2_ receptors, respectively [107]. They synthesized sixteen new derivatives, seven of which were characterized by a benzoylpiperidine fragment while still maintaining the fundamental chemical features of the lead compound **77**.

The most active compound of the newly synthesized 4-aroylpiperidines was compound **78**, with a *K*_i_ value of 1.40 nM for the σ1 receptor and 854 nM for the σ2 receptor, and a notable selectivity ratio (*K*_i_ σ_1_/σ_2_ = 610) compared with lead compound **77**. These results suggested that the spirofused ring of compound **77** was not essential for inhibiting the σ1 receptor. Moreover, computational studies clarified that the interaction with the σ_1_ receptor was driven by hydrophobic interactions.

#### 3.2.4. AChE Inhibitors

Alzheimer’s disease (AD) is a neurodegenerative disease whose etiology is still not clearly defined. One of the most investigated pathophysiological hypotheses is the cholinergic one: the deficiency of the neurotransmitter ACh in neurons contributes significantly to the cognitive decline observed in AD patients [108]. Among the pharmacotherapies used for the management of AD, the employment of acetylcholinesterase inhibitors (AChEI), able to block ACh metabolism and thus increase both the level and duration of the neurotransmitter action, represents a reasonable approach to treating AD and other forms of dementia.

**Figure 19:**
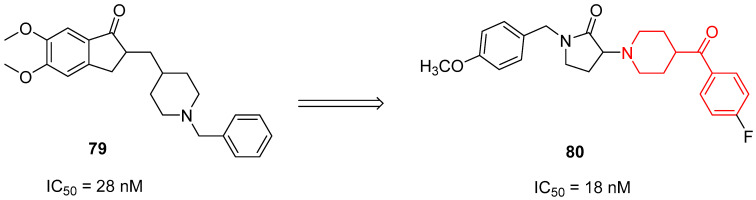
Design and structure of Donezepil (**79**) and the AChEI **80** possessing a benzoylpiperidine fragment (in red).

In 2020, Gupta and collaborators decided to optimize the structure of Donezepil (compound **79**, Figure 19), an AChEI commonly used in the treatment of AD patients, on the basis of SAR studies present in the literature [20]. They synthesized several new AChEIs characterized by different structures, and the most promising of the pyrrolidine-2-one series was the benzoylpiperidine-based compound **80** (IC_50_ = 18 nM, Figure 19).

Benzoylpiperidine **80** displayed the highest neuroprotective effect, compared with Donezepil, by substantially reducing escape latency in the Morris water maze model. Furthermore, biochemical assays to evaluate the acetylcholinesterase (AChE) levels, lipid peroxidation, nitrite levels, and oxidative stress in mouse brain homogenates treated with compound **80** demonstrated its ability to restore maximum normalcy of the biochemical mediators. Taken together, this evidence supports the possibility of utilizing AChEIs for the management of AD patients.

Unluckily, further chemical optimization of the lead compound pyrrolidine-2-one AChEI **80** did not lead to the identification of new benzoylpiperidine derivatives, but the best results were obtained by molecules belonging to other chemical classes [109].

#### 3.2.5. Other Neuroprotective Agents

**Figure 20:**
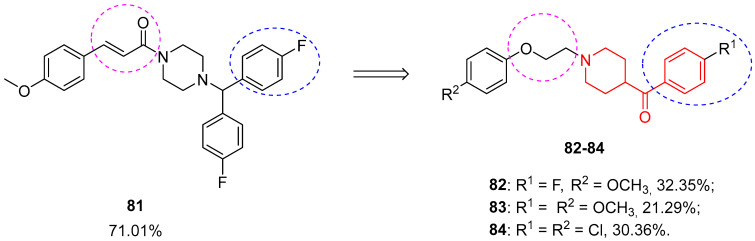
Structure of compound **81** and of neuroprotective agents **82**–**84** possessing a benzoylpiperidine fragment (in red). Percentage refers to cell viability when compounds are tested at a 0.1 µM concentration.

Very recently, Zhong and co-workers synthesized a new class of benzoylpiperidines with neuroprotective effects starting from the lead compound **81** (Figure 20), previously identified by the same research group [42].

All 37 synthesized benzoylpiperidines were tested both in vitro and in vivo to assess their neuroprotective activity. In MTT assays, all the compounds exerted a neuroprotective effect on glutamate-induced PC12 cells, with the best results in terms of percentage of cell viability for **82**, **83**, and **84** (Figure 20) with values of 32.35, 21.29, and 30.36%, respectively, when tested at a 0.1 µM concentration. Their neuroprotective activity was also confirmed by in vivo experiments on bilateral occlusion of the common carotid arteries (BCCAO) in Kunming mice. At all five tested doses, compounds **82**, **83**, and **84** remarkably augmented the survival of mice with acute cerebral ischemia. This study paved the way for the development of new neuroprotective agents to treat cerebral ischemic stroke based on a benzoylpiperidine fragment.

### 3.3. Tuberculosis and Parasites

#### 3.3.1. Antitubercular Agents

Tuberculosis (TB) is an infectious disease caused by *Mycobacterium tuberculosis* (Mtb). TB remains a serious health threat worldwide, and it is considered the second leading cause of death from contagious disease in the world. A combination therapy method (first-line drugs: rifampin, isoniazid, pyrazinamide, and ethambutol) developed by the World Health Organization (WHO) is one of the most efficient weapons against TB. However, its success rate is struggling to reach the target of 85% [110,111]. In addition, multi-drug-resistant (MDR-TB) and extensively drug-resistant (XDR-TB) strains of Mtb strongly impact the first-line regimen activity of Mtb [112]. Therefore, to improve the efficacy and tolerability of TB treatment, shorten treatment, and simplify treatment, new drugs are necessary.

**Figure 21:**
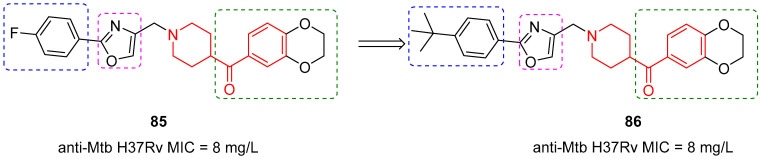
Structure of Mtb inhibitors **85**, **86**. The benzoylpiperidine fragment is highlighted in red.

In this scope, Li et al. performed a high-throughput screening (HTS) on a 45,000-compound library from the National Center for Screening New Microbial Drugs to identify a new small Mtb inhibitor by using *Mycobacterium smegmatis* mc^2^-155 as a model [43]. *Mycobacterium smegmatis* is a model mycobacterium that grows rapidly and is not pathogenic; indeed, it can be utilized as a substitute for Mtb to avoid using highly pathogenic and slow-growing Mtb in the early drug screening process. HTS allowed the identification of compound **85** (Figure 21) as a potent *Mycobacterium smegmatis* mc^2^-155 inhibitor with an in vitro minimal inhibitory concentration (MIC) of 0.3 mg/L. Compound **85** also showed good inhibitory activity against Mtb H37Rv with a MIC of 8 mg/L in vitro. Based on this preliminary assay, the SAR of compound **85** was investigated to develop new derivatives with lower cytotoxicity and increased potency against Mtb. One of the most representative Mtb inhibitors in this series was benzoylpiperidine **86** (Figure 21). The benzoylpiperdine fragment (highlighted in red, Figure 21), the 1,3-oxazole ring (magenta dashed square, Figure 21), and the benzoyl moiety (green dashed square, Figure 21) of **85** were maintained, while the principal change was made to the distal phenyl ring, by introducing a *tert*-butyl group instead of the fluorine atom (blue dashed square, Figure 21).

Compound **86** was subjected to in vitro evaluation against replicating Mtb H37Rv, and it showed potent anti-TB activity with a MIC of 8 mg/L. In addition, cellular toxicity was investigated by using HEK293 cells, and the results showed that both **85** and **86** were not cytotoxic (IC_50_ > 150 µM in all cases). Finally, **86** anti-Mtb activity in MDR-Mtb and XDR-Mtb strains was very encouraging (2 mg/L and 1 mg/L, respectively) and proposed a novel mechanism of action for these compounds.

#### 3.3.2. Antiparasitic Agents

*Giardia lamblia* (also known as *G. duodenalis* or *G. intestinalis*) is a single-cell parasite that populates the small intestine. This etiologic agent causes a re-emerging infectious disease called giardiasis [113]. This disease is manifested by chronic diarrhea and poor nutritional absorption, and its transmission takes place through the fecal-oral route. For many years, metronidazole has been the drug of choice for the pharmacological treatment of giardiasis, but recently, the most commonly used drugs include benzimidazoles, nitrofuranes, quinacrine, and macrocyclic lactones [114,115,116]. However, there is an impelling need to find new antiparasitics against G. lamblia because of the increasing therapeutic failure due to low compliance with drug therapy, re-infestation, and drug resistance to metronidazole and its related derivatives [117].

**Figure 22:**
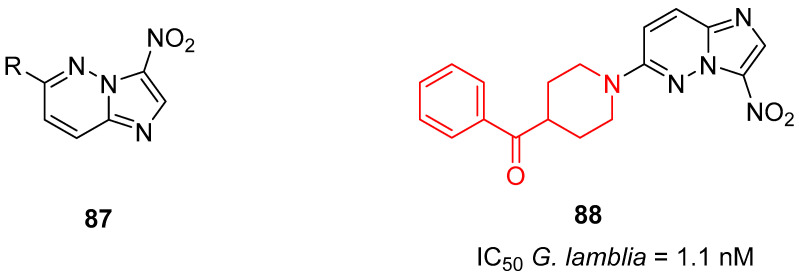
General structure of nitroimidazopyridazine antiparasitics **87** and structure of **88** (benzoylpiperidine fragment in red).

Very recently, Zheng and colleagues discovered 2-nitroimidazo[1,2-*b*]pyridazine as a novel scaffold to develop antiparasitics against *G. lamblia* with sub-nanomolar activity [21]. The design of these compounds originated from broad-spectrum nitroimidazopyridazine antiparasitics with general scaffold **87** (Figure 22) [118]. Among all the synthesized compounds, one of them (compound **88**, Figure 22) possessed a benzoylpiperidine fragment in its structure.

Compound **88** possessed a very high anti-parasite potency against *G. lamblia* (IC_50_ of 1.1 nM) without significant cytotoxicity against human lung fibroblasts MRC-5 (IC_50_ > 6.4 × 10^4^ nM). In addition, compound **88** showed suitable calculated physiochemical properties in terms of cLog*P* (3.2), polar surface area (tPSA of 93.6 Å^2^), and molecular weight (351.4 g/mol).

### 3.4. Metabolic Syndrome, Diabetes, and Lipid-Related Diseases

#### 3.4.1. SCD-1 Inhibitors

Stearoyl-CoA desaturase-1 (SCD-1) is an enzyme responsible for the production of monosaturated fatty acids from their saturated fatty acid precursors. This rate-limiting enzyme was found to be fundamental in the regulation of lipid metabolism and in the control of body weight, and its deficiency results in resistance against obesity, reduces liver steatosis in rodents, and diminishes the production of cholesterol ester and triglycerides [119,120,121,122,123,124]. Indeed, SCD-1 could represent a feasible target for the treatment of metabolic syndrome and other lipid-related diseases.

**Figure 23:**
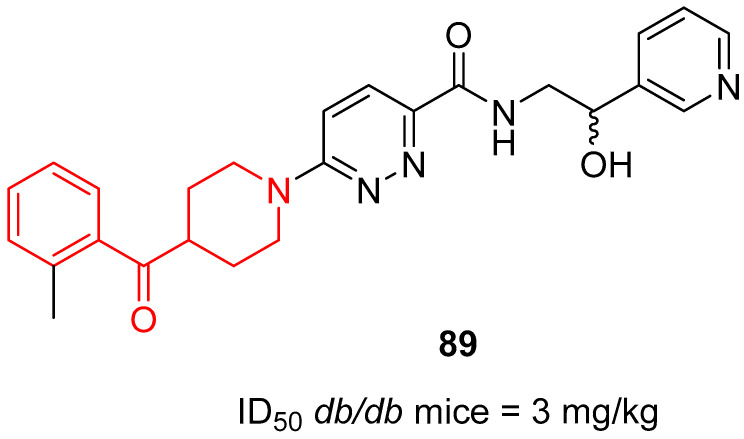
Structure of the most active SCD-1 inhibitor **89** developed by the research group of Ohsumi, possessing a benzoylpiperidine fragment (in red).

In 2010, the research group of Ohsumi optimized a well-known piperazine-based SCD-1 inhibitor, thus discovering a new class of SCD-1 inhibitors characterized by a benzoylpiperidine fragment [61]. The most active compound of this series was *N*-(2-hydroxy-2-(pyridin-3-yl)ethyl)-6-(4-(2-methylbenzoyl)piperidin-1-yl)pyridazine-3-carboxamide **89** (Figure 23), with a methyl group in the 2-position of the benzoylpiperidine moiety and a 3-pyridyl ring on the other side of the molecule.

Derivative **89** showed an ID_50_ of 3 mg/kg in studies on *db*/*db* mice, the most widely used mouse model of obesity and type 2 diabetes mellitus. Regarding ADME and pharmacokinetic properties, benzoylpiperidine **89** was soluble at neutral pH and metabolically stable. It also showed increased plasma concentration and a higher bioavailability in C57BL/6J mice after oral administration compared with its analogue, which possessed a simple phenyl ring instead of the 3-pyridyl one. SCD-1 inhibitor **89** was also tested in Zucker fatty rats, another commonly used animal model of obesity, to define its efficacy in vivo, where it diminished plasma triglyceride levels in a dose-dependent manner after a 7-day oral administration. Taken together, all these results highlighted that benzoylpiperidine **89** could be further modified to develop novel and more potent SCD-1 inhibitors useful in the treatment of metabolic syndrome.

#### 3.4.2. AMPK Activators (Complex I Inhibitors)

Inhibition of Complex I (see Section 3.1.3) causes the activation of 5′-AMP-activated protein kinase (AMPK), an important key sensor of cellular energy status, thus resulting in the mobilization of nutrient uptake and catabolism to generate energy through mitochondrial ATP production. Hence, the activation of AMPK via Complex I inhibition is an intriguing option to cure diabetes and other metabolic syndromes [125,126].

**Figure 24:**
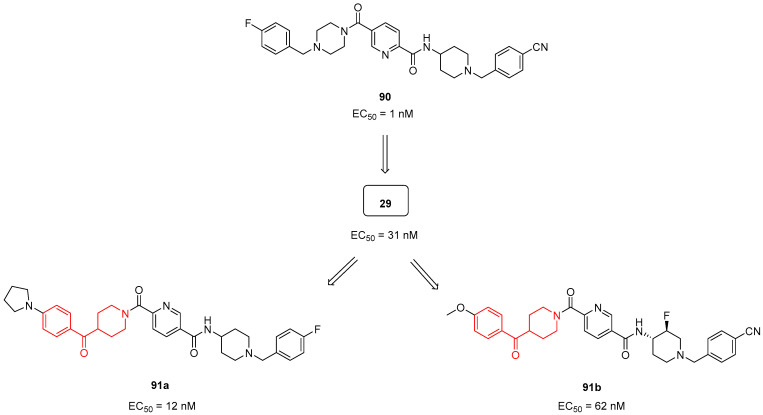
Structures of AMPK activators **90**–**91b**. EC_50_ values refer to AMPK activation in HepG2 cells. The benzoylpiperidine fragment is highlighted in red.

For this purpose, in 2013, Hitoshi and colleagues identified a new Complex I inhibitor, **29** (Section 3.1.3. Figure 7), able to indirectly activate AMPK [22]. The synthesis of this compound was previously reported in a patent and mentioned in Section 3.1.3. [127]. Compound **29** proved to regulate mitochondrial function in vitro and to improve metabolic parameters in diabetic animals in vivo. In 2022, investigations were performed to evaluate the SAR of AMPK activator **90** (Figure 24) and to synthesize new derivatives as potential anti-diabetic agents [128]. Thanks to SAR studies, they found out that the replacement of the piperazine ring of **90** with a benzoylpiperidine portion as in compound **29** brought about a substantial increase in human liver microsomial stability (t_1/2_ of 71 min of **29** versus 15 min of **90**) and no significant inhibition of cytochrome P450 (CYP450) isoform 3A4. Moreover, **29** was less able to inhibit the human ether-à-go-go related gene (hERG) potassium channel compared with lead **90**, thus reducing the risk of fatal cardiac arrhythmia. The researchers continued to optimize the structure of **29** by exploring the substituents of the two distal phenyl rings, thus obtaining a series of analogues exemplified by compound **91a** (Figure 24). However, the pharmacological evaluation was focused on **29**, which proved to activate AMPK in vivo after oral administration at 10 mg/kg by reducing blood glucose levels in a *db*/*db* diabetes mouse model. These results were confirmed in the diet-induced obesity (DIO) animal model, where **29** improved glucose handling when dosed in chow at levels >5 mg/kg.

Very recently, Hitoshi and collaborators further modified **29**, thus obtaining a new promising AMPK activator: compound **91b** (Figure 24) [129]. Compound **91b** differs from **29** because of the presence of a fluorine atom at position 3 of the piperidine ring. The *trans*-3-fluoropiperidine analogue **91b** exerted a notable on-target activation of AMPK with EC_50_ values of 0.062 and 0.161 µM on both HepG2 and mouse muscle (C2C12) cells, respectively. The introduction of a 3-fluoro substituent in compound **91b**, by attenuating the basicity of the piperidine moiety, significantly lowered the hERG inhibition compared with **29** (IC_50_ values of 69 and 4.7 µM, respectively). Furthermore, the 3-fluoro substituent of **91b** profoundly reduced in vivo rat clearance from 150 (value for lead **29**) to 17 mL/min/kg. In rat PK studies, AMPK activator **91b** showed good oral bioavailability (55% F). *trans*-3-Fluoropiperidine **91b** was further tested in vivo in a *db*/*db* model of type II diabetes, where it showed a dose-dependent improvement in glucose handling together with the reduction of fasting blood glucose and insulin levels.

### 3.5. Cardiovascular Diseases

#### 3.5.1. hERG K^+^ Ligands

The human ether-à-go-go related gene (hERG) K^+^ channel (also known as Kv11.1 or KCNH2) is of raising interest in the pharmaceutical industry for drug development because many drugs block it, causing QT interval prolongation, which is a risk factor for ventricular arrythmia and fibrillation that consequently lead to *torsades de pointes*, a type of polymorphic ventricular tachycardia, and then death. Indeed, different drugs, such as astemizole, sertindole, and cisapride, were withdrawn from the market because of their cardiotoxic effects due to hERG K^+^ blockade. Therefore, the prediction of the cardiotoxicity determined by off-target hERG K^+^ blockade became crucial in the early stage of drug development to avoid failure in later human clinical trials or, even worse, to avoid the withdrawal of the drug from the market [130]. However, hERG K^+^ can also be exploited for therapeutic purposes; class III antiarrhythmics accomplish their function by interacting with hERG K^+^ and thus stabilizing the heart rhythm.

**Figure 25:**
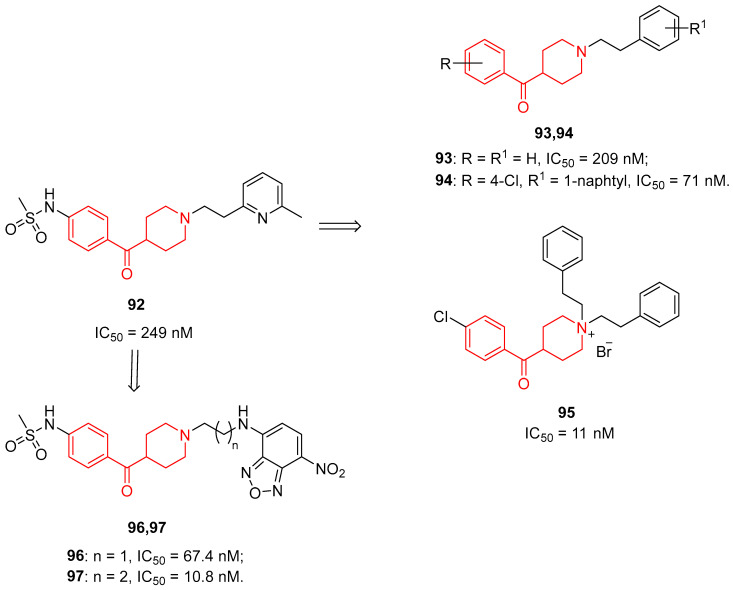
hERG K^+^ ligands possessing a benzoylpiperidine fragment (in red).

Considering this background, Professor IJzerman’s research group decided to investigate compounds targeting hERG K^+^ by modifying the structure of the class III antiarrhythmic drug **92** (Figure 25) and then evaluating the SAR of the newly developed compounds [44].

The affinity of these compounds for the hERG K^+^ channel was evaluated in a [^3^H]-astemizole radioligand binding assay by using HEK293 cell membranes characterized by the overexpression of this channel. The SAR analysis suggested that the complete removal of the sulfonamide group in the *para* position of the benzoyl moiety, such as in compound **93** (Figure 25), caused a significant decrease in hERG K^+^ affinity. On the other side of the molecule, the introduction of bulkier aromatic groups, such as in compound **94** (Figure 25), proved to be well tolerated, thus suggesting that the cavity of the binding site is quite wide to host bulky groups. Contrarily, the shift from a tertiary to a quaternary amine led to a notable increase in hERG K^+^ affinity (a representative example is compound **95**, Figure 25). However, the most useful strategy to prevent hERG K^+^ blockade was the substitution of the central piperidine ring with a piperazine ring. Taken together, these results offered a very helpful insight for designing novel potential drugs with a reduced cardiotoxicity risk due to hERG K^+^ blockade, but also for developing new class III antiarrhythmics endowed with a better affinity for hERG K^+^. The role of the aromatic group and the basic nitrogen of the benzoylpiperidine fragment in the increment of hERG K^+^ affinity was lately confirmed in other works from the same research group [23,131].

Other evidence highlights that the hERG K^+^ channel is involved in cancer progression, thus making it a reliable target for cancer treatment [132]. At present, the advancement of new, safe, and cost-effective fluorescent methods to better analyze the hERG K^+^ channel at a molecular level is of great importance. To this purpose, Li and coworkers developed new nitrobenzoxadiazole (NBD)-based environment-sensitive fluorescent probes for the hERG K^+^ channel [25]. The small NBD fluorophore was chosen because it acts as a recognition group in the hydrophobic interaction with Tyr652 and/or Phe656 residues of the hERG K^+^. Moreover, the researchers chose the major interacting part of **92**, that is, the benzoylpiperidine moiety, as the other recognition group of the probe. Indeed, probes **96** and **97** possess a benzoylpiperidine frame in their structures (Figure 25). Probe **97** displayed a potent affinity for hERG K^+^ with an IC_50_ value of 10.8 nM and a *K*_i_ value of 6.08 nM, which are quite acceptable when compared with those of the reference compound astemizole. In conclusion, these probes could be used in physiological and pathological studies of the hERG K^+^ channel and could represent a feasible screening system for this channel.

#### 3.5.2. Factor Xa Inhibitors

Factor Xa (FXa) is a serine protease of the blood coagulation cascade involved in the production of thrombin by both intrinsic and extrinsic pathways. In particular, those molecules that are able to inhibit factor Xa may be used as anti-thrombotic agents [133,134].

**Figure 26:**
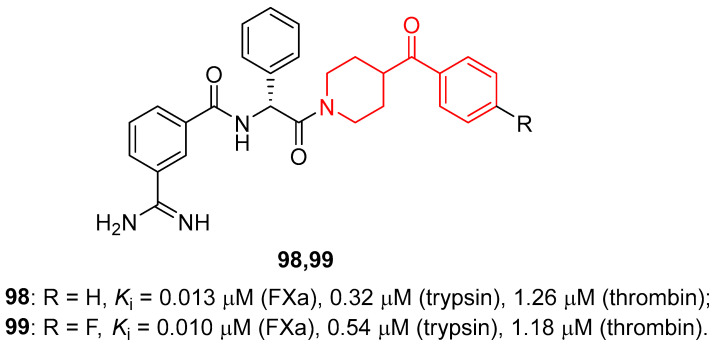
Structure of the most representative factor Xa inhibitors **98** and **99** identified by Jones et al. [26,135] possessing a benzoylpiperidine fragment (in red).

Aiming at developing new anticoagulants, Jones and co-workers used an informatic tool for the virtual screening of libraries, to identify new FXa inhibitors [26,135]. This study led to the design and synthesis of different libraries of compounds, which were then tested to evaluate their ability to inhibit FXa. The most promising derivatives of this study were compounds **98** and **99** (Figure 26), with *K*_i_ values in the low nanomolar range of 13 and 10 nM, respectively. Furthermore, compounds **98** and **99** also demonstrated good selectivity for FXa compared with other related enzymes: trypsin (*K*_i_ values of 0.32 and 0.54 μM, respectively) and thrombin (*K*_i_ values of 1.26 and 1.18 μM, respectively). Finally, crystallographic studies using trypsin as the model enzyme, considering its similarity to FXa, supported the binding mode of compound **98** in the FXa binding site and highlighted the importance of the lipophilic interaction between the benzene ring of the *D*-phenylglycine of the ligand and the disulphide pocket of the enzyme.

### 3.6. Other Disorders

#### Beta-Adrenoceptor Ligands

Today, β-adrenoceptor ligands are commonly used to treat various disorders. For example, β2-adrenergic receptor selective agonists are employed for treating asthma and other bronchospastic diseases, but they are also used as tocolytics in cases of preterm labor [136,137,138,139,140].

**Figure 27:**
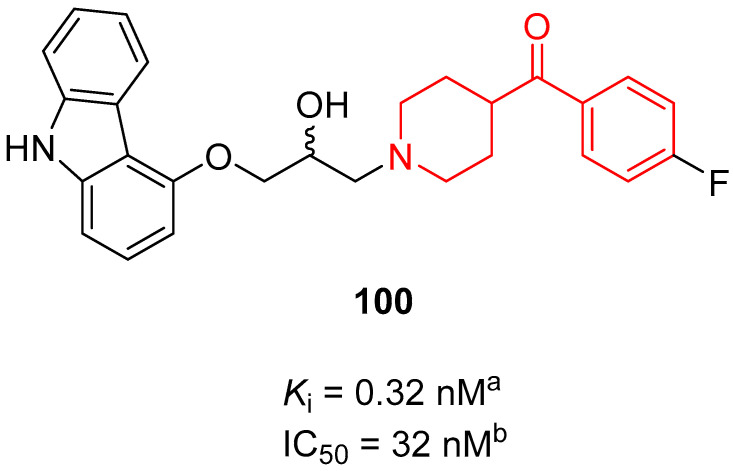
Structure of the most representative β2-adrenoceptor ligand **100** identified by Tasler et al. [141], possessing a benzoylpiperidine fragment (in red). ^a^ hβ2-adrenergic affinity in binding assays. ^b^ hβ2-adrenergic antagonistic effect in functional assays.

In 2010, Tasler and co-workers identified new β-adrenoceptor ligands through a vHTS approach [141]. This investigation resulted in the identification of benzoylpiperidine **100** (Figure 27), endowed with the best affinity for human β2-adrenergic (hβ2-AR) receptors in the picomolar range (*K*_i_ value of 0.32 nM) and characterized by good antagonistic activity at hβ2-AR (IC_50_ value of 32 nM). This carbazol-4-yloxy derivative was also subjected to further investigation to better analyze its pharmacokinetic profile. Compound **100** was stable in artificial gastric juice, simulated intestinal fluids, and human plasma for 6 h; moreover, it demonstrated to be stable in aqueous media and showed a good permeability in the PAMPA assay. Finally, its toxicity profile was favorable, with ED_50_ values of 9 and 19 μM in PBMC and HepG2 tests, respectively, thus making benzoylpiperidine **100** a valid starting point for the identification and development of new β2-adrenoceptor antagonists for treating different disorders.

### 3.7. Diagnostic Agents

#### 3.7.1. VAChT Ligands for PET Brain Imaging

The loss of cholinergic neurons and synapses in the brain is the main cause of the progressive diminution in cognitive functions characteristic of neurodegenerative diseases. Indeed, inhibitors of AChE able to augment the levels of ACh, such as rivastigmine and donezepil, are widely used in the treatment of this type of pathology [142]. The vescicular acetylcholine transporter (VAChT) is the protein responsible for the transport of newly synthesized ACh into synaptic vescicules [143], and it has been demonstrated to be a feasible molecular marker of the cholinergic system. In this regard, PET radiotracers able to quantify VAChT levels in vivo could be utilized as biomarkers to examine alterations in cholinergic functions within the brains of animals or humans undergoing therapy.

**Figure 28:**
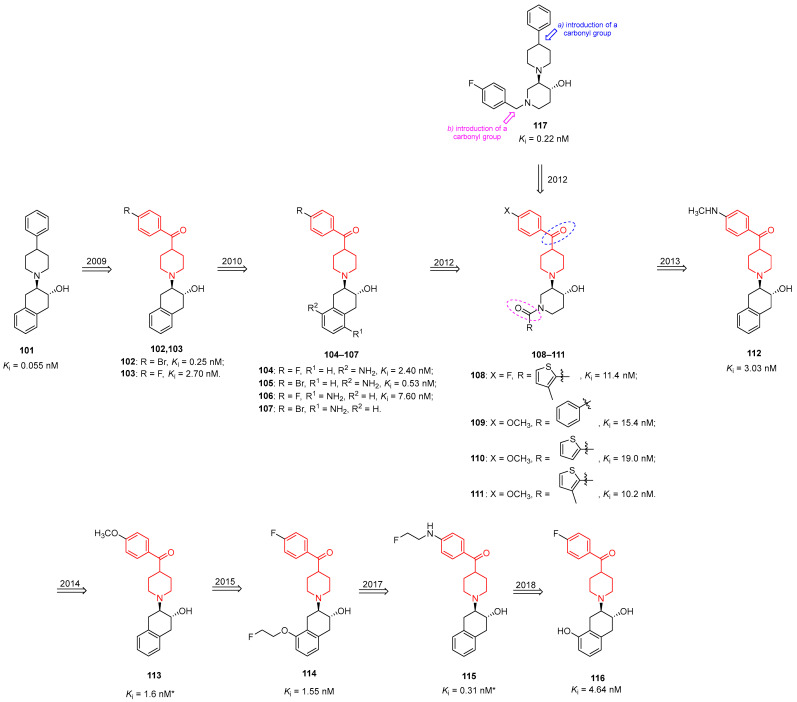
Structures of the VAChT ligands as PET radiotracers identified by Tu et al. [51] possessing a benzoylpiperidine fragment (in red). * *K*_i_ value refers to the *minus* isomer.

In 2009, Tu and collaborators synthesized a new class of ^18^F-labeled PET tracers for the imaging of VAChT, characterized by a benzoylpiperidine moiety [51]. They replaced the phenyl ring of the well-known VAChT ligand benzovesamicol (compound **101**, Figure 28) with a *para*-substituted benzoyl moiety, thus generating analogues **102** and **103** (Figure 28).

In vitro binding studies highlighted that compound **102**, characterized by a bromine atom in the *para* position of the benzoyl moiety, was endowed with the best affinity for VAChT (*K*_i_ = 0.25 nM), whereas compound **103** showed a moderate affinity for VAChT (*K*_i_ = 2.70 nM). Moreover, compounds **102** and **103** were also demonstrated to be selective for VAChT compared with σ_1_ and σ_2_ receptors. Benzoylpiperidines **102** and **103** could therefore be utilized as PET radiotracers when labeled with ^76^Br and ^18^F, respectively. Although compound **102** was more potent and selective than compound **103**, ^76^Br is considered less optimal for PET imaging due to its radionuclide decay characteristics when compared with ^18^F. In fact, PET radiotracers incorporating ^18^F produce superior-quality images in comparison to those incorporating ^76^Br. For this reason, Tu et al. [51] proceeded with the radiosynthesis of (±)-*trans*-[^18^F]-**103**, (+)-*trans*-[^18^F]-**103**, and (−)-*trans*-[^18^F]-103, considering that in in vitro evaluation, the pure enantiomer (−)-*trans*-**103** showed a better VAChT/σ selectivity compared to the racemic mixture **103**. In vivo assays of radiolabeled (±)-*trans*-[^18^F]-**103**, (+)-*trans*-[^18^F]-**103**, and (−)-*trans*-[^18^F]-**103** highlighted that (−)-*trans*-[^18^F]-**103** was more potent than its enantiomer; moreover, the *minus* isomer was also retained in target regions of the brain, following a similar pattern to the density of VAChT in this organ. In addition, MicroPET imaging studies in a male rhesus macaque treated with (−)-*trans*-[^18^F]-**103** highlighted that the probe easily penetrated the brain, and its distribution pattern reflected that of the VAChT in the brain. Taken together, these results demonstrated that benzoylpiperidine (−)-*trans*-[^18^F]-**103** is a promising PET radiotracer for imaging the VAChT and thus studying cholinergic innervations in the brain.

One year later, the same research group conducted a structure-activity relationship (SAR) investigation on a new class of VAChT ligands aimed at finding compounds with an increased affinity and selectivity for this transporter [53]. The results of this study were compared with those of the previous work [51]. Among the newly synthesized VAChT ligands, regioisomeric pairs **104**, **105** and **106**, **107** were characterized by a benzoylpiperidine portion (Figure 28). In in vitro binding studies, no significant improvements were produced by the insertion of the amino susbtituent, as demonstrated by the *K_i_* values of compounds **103** and **104** (*K*_i_ of 2.70 and 2.40 nM, respectively) and compounds **102** and **105** (*K*_i_ of 0.25 and 0.53 nM, respectively). However, there was a distinction in affinity observed between the regioisomeric pairs **104** and **106**, with *K*_i_ of 2.40 and 7.60 nM, respectively, thus demonstrating the preferential position of the amino group. Affinity values for regioisomer **107** were not reported in this work. Finally, benzoylpiperidines **104**, **105** were endowed with a high selectivity for VAChT versus σ_1_ and σ_2_ receptors. In this regard, compounds **104**, **105** could be radiolabeled to provide PET probes for studying VAChT expression in the brain.

The exploration of new VAChT ligands for PET brain imaging continued, and in 2012, the researchers decided to modify the structure of fluorobenzyltrozamicol (compound **117**, Figure 28) by: (*a*) introducing a carbonyl group between the aromatic and piperidine rings, taking into account the previously developed VAChT ligands (modification in blue, Figure 28) [51,53]; (*b*) substituting the benzyl group of tertiary amines with a benzoyl group to make tertiary amides (modification in magenta, Figure 28). They identified 18 new compounds, four of which, compounds **108**–**111** (Figure 28), demonstrated encouraging results in in vitro bioactivity evaluation [54]. *Trans* racemates **108**–**111** showed high affinity for VAChT (*K*_i_ values ranging from 10.2 to 19.0 nM) and also increased selectivity for VAChT compared with σ_1_ and σ_2_ receptors (selectivity VAChT/σ_1_ ratio > 374 and selectivity VAChT/σ_2_ ratio > 315). Noteworthy, substitution on the *para* position of the benzoylpiperidine fragment with an electron-rich methoxy group (**111**, *K*_i_ = 10.2 nM) led to an increase in terms of potency compared with its *p*-fluoro-substituted analogue (**108**, *K*_i_ = 11.4 nM). The four tested compounds also showed good lipophilicity properties with a Log*P* in the range of 2–3 (except for compound **109**), thus suggesting their penetration through the BBB and inciting their radiolabeling with ^11^C or ^18^F to confirm their possible use as PET radiotracers.

The optimization of the structure of the above-mentioned VAChT PET tracer to increase affinity for VAChT led to a new series of derivatives, which were synthesized in 2013 [55]. Among them, compound **112** (Figure 28) turned out to be the most promising one, with a *K*_i_ value of 3.03 nM. Derivative **112** also exhibited a selectivity ratio for VAChT versus σ receptors greater than 100-fold. To validate the feasibility of **112** as a PET radiotracer, *trans* racemate **112** was resolved thanks to the chiral HPLC technique to obtain the *minus* and *plus* isomers. The hypothesis that the ligand binding to VAChT is stereoselective was in accordance with the fact that, also in this work, the *minus* isomer showed a higher potency for VAChT compared with the racemate and the *plus* isomer. Indeed, (−)-*trans***-112** was more potent than the racemate and (+)-*trans***-112**, with *K*_i_ values of 0.78, 3.03, and 19.0 nM, respectively. The radiolabeled (−)-*trans*-[^11^C]-**112** was synthesized and tested in vivo in rats and monkeys, where it proved to penetrate the BBB with a massive accumulation in the striatum, the brain area in which the VAChT is mainly localized. The tissue-time activity curves post-injection of (−)-*trans*-[^11^C]-**112** in the brain of a male Cynomolgus monkey highlighted its advantageous pharmacokinetic properties. Further in vitro and in vivo investigations validated (−)-*trans*-[^11^C]-**112** as a potential candidate for assessing the level of VAChT in the brain [56].

Always focusing on ^11^C-labeled VAChT tracers, the same research group synthesized, resolved, and radiolabeled derivative **113** (Figure 28) [58]. The biological profile of (−)-*trans*-**113** was evaluated in another paper [57], and it showed a higher potency for VAChT compared with (+)-*trans*-**113** (*K*_i_ values of 1.6 and 34.0 nM, respectively). Additionally, (−)-*trans*-**113** was selective for VAChT versus σ_1_ and σ_2_ receptors, with a selectivity VAChT/σ_1_ ratio greater than 35 and a selectivity VAChT/σ_2_ ratio of 1600. The biodistribution and regional brain uptake of (−)-*trans*-[^11^C]-**113** were evaluated in rats and nonhuman primates (NHP). The results demonstrated that (−)-*trans*-[^11^C]-**113** possessed valuable brain penetration, high target-to-nontarget ratios, and favorable washout pharmacokinetics in both animal models.

Taking into consideration that the half-life of ^18^F (t_1/2_ = 109.8 min) allows longer scan sessions than those with ^11^C (t_1/2_ = 20.3 min), thus generating higher target-to-reference ratios, the researchers wanted to introduce a fluorine atom for ^18^F-radiolabeling in the previously reported carbonyl-containing benzovesamicol analogs. Moreover, they optimized the structure of benzovesamicol analogs by adopting the following strategies: *a)* insertion of PEGylated chains in the benzoylpiperidine moiety, which can improve clearance kinetics by decreasing lipophilicity; *b)* introduction of a fluoroethoxy group in the tetralin moiety to further improve affinity and selectivity for VAChT versus the σ receptors [59]. Among the newly fluorinated benzovesamicol analogues, compound **114** (Figure 28), possessing a 2-fluoroethoxy group in the 5-position of the tetralin moiety, was one of the most interesting ones, with a *K*_i_ value for VAChT of 1.55 nM and a remarkable selectivity for VAChT compared with σ_1_ and σ_2_ receptors. The *trans* racemate **114** was resolved by chiral HPLC, and the *minus* and *plus* isomers were isolated. In accordance with the stereoselectivity of VAChT binding, the *minus* isomer (−)-*trans*-**114** was more active compared with the *plus* isomer and the racemate, with a *K*_i_ value for VAChT of 0.59 nM, and it also showed a high selectivity versus σ receptors (>10,000-fold) and a good lipophilicity (calculated Log*P* of 3.45). For these reasons, the derivative (−)-*trans***-114** was chosen for radiolabeling with ^18^F and for further biological investigations. In vivo studies in rodents and NHPs demonstrated that (−)-*trans*-[^18^F]-**114** was able to cross the BBB and accumulate in the striatum. (−)-*trans*-[^18^F]-**114** also possessed valid biodistribution properties and good metabolic stability in vivo, thus assigning a promising future to (−)-*trans*-[^18^F]-**114** as a perspective PET radiotracer for brain imaging.

Further efforts at the structural optimization of benzovesamicol analogues led to the identification of compound (±)-*trans*-**115** (Figure 28), a benzoylpiperidine analogue with a 2-fluoroethylamino group in *para* position on the benzoyl moiety instead of the methoxy group present in compound **113** [60]. Chiral resolution of *trans* isomers was performed, and the binding affinity of each enantiomer was determined and compared with the racemate. As expected, the most potent and selective compound was the *minus* isomer (−)-*trans*-**115** (*K*_i_ value of 0.31 nM for VAChT), which was then radiolabeled with ^18^F and tested in NHPs as the previously developed PET tracers. The microPET imaging results demonstrated that (−)-*trans*-[^18^F]-**115** penetrated the BBB and mainly accumulated in the VAChT-enriched striatum brain region. (−)-*trans*-[^18^F]-**115** possessed favorable kinetic properties in the NHP brain, and it deserved further investigation to validate its possible use as an efficient diagnostic agent.

The most recent work published by this research group about VAChT imaging agents concerned the further optimization of compound **114**, previously developed in 2015, in which the 5-(2-fluoroethoxy) group was replaced by a free hydroxyl group (compound **116**, Figure 28) [52]. Racemic compound (±)-*trans*-**116** had a good binding affinity (*K*_i_ value of 4.64 nM) for VAChT and very low binding affinities for *σ*1 receptors and *σ*2 receptors (*K*_i_ values of 8640 and 1851 nM, respectively). The free hydroxyl group in the tetralin moiety of compound **116** lowered the hydrophilicity (Log*P* = 1.78) compared with **114** (Log*P* = 3.45). The *trans* racemic mixture of **116** was resolved, and the *minus* enantiomer was radiolabeled with ^18^F. (−)-*trans*-[^18^F]-**116** displayed fast clearance kinetics from the target striatal region of the brain in preliminary imaging results on NHP when compared with (−)-*trans*-[^18^F]-**114**. Despite this, its lower target-to-non-target ratio could make its development as a PET radiotracer for quantitative measurement of the VAChT in the human brain difficult.

**Figure 29:**
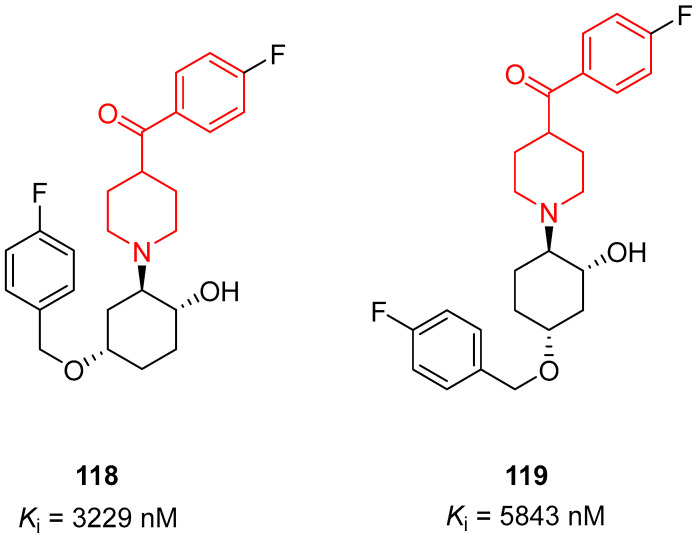
Structure of benzoylpiperidine (fragment highlighted in red) VAChT ligands as a diagnostic developed by Barthel et al. [27].

Another study from the University of Leipzig reported the synthesis of a library of vesamicol and benzovesamicol analogues and the biological evaluation of their VAChT affinity and selectivity versus σ receptors [27]. Among the several synthesized compounds, two of them possessed the benzoylpiperidine portion (compounds **118** and **119**, Figure 29), such as the previously described compounds. However, in this case, the introduction of the benzoylpiperidine moiety did not substantially improve the binding affinity for VAChT since compounds **118** and **119** showed weak binding affinities (*K*_i_ values of 3229 and 5843 nM, respectively).

#### 3.7.2. 5-HT_2A_ Ligands for PET or SPECT Brain Imaging

**Figure 30:**
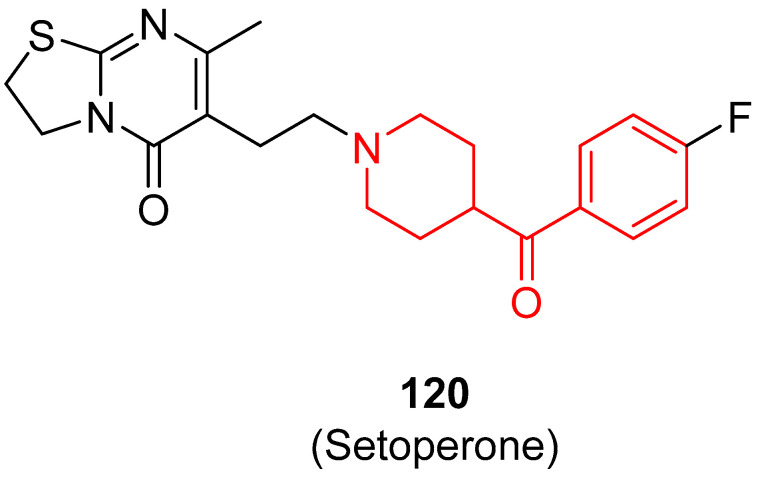
Structures of the 5-HT_2A_ ligand Setoperone (**120**). The benzoylpiperidine fragment is highlighted in red.

Imaging 5-HT_2A_ receptors in vivo is very useful to evaluate receptor occupancy, set the dosage of a drug, investigate the cerebral and plasma pharmacokinetics, and, finally, hopefully, foresee the efficacy of the treatment with antipsychotics. With this purpose, [^18^F]-**2**, [^11^C]-(+)-**54**, and [^18^F]-**120** (Setoperone (**120**), Figure 30), three selective 5-HT_2A_ ligands, were developed as diagnostics [144,145,146].

Taking into account the limitations of these diagnostics ([^18^F]-**2** possesses only a 20-fold higher affinity for 5-HT_2A_ receptors compared with 5-HT_2C_, whereas [^11^C]-(+)-**54** is endowed with a short half-life), the development of more potent, selective, and long-life radioligands is a thrilling research field.

**Figure 31:**
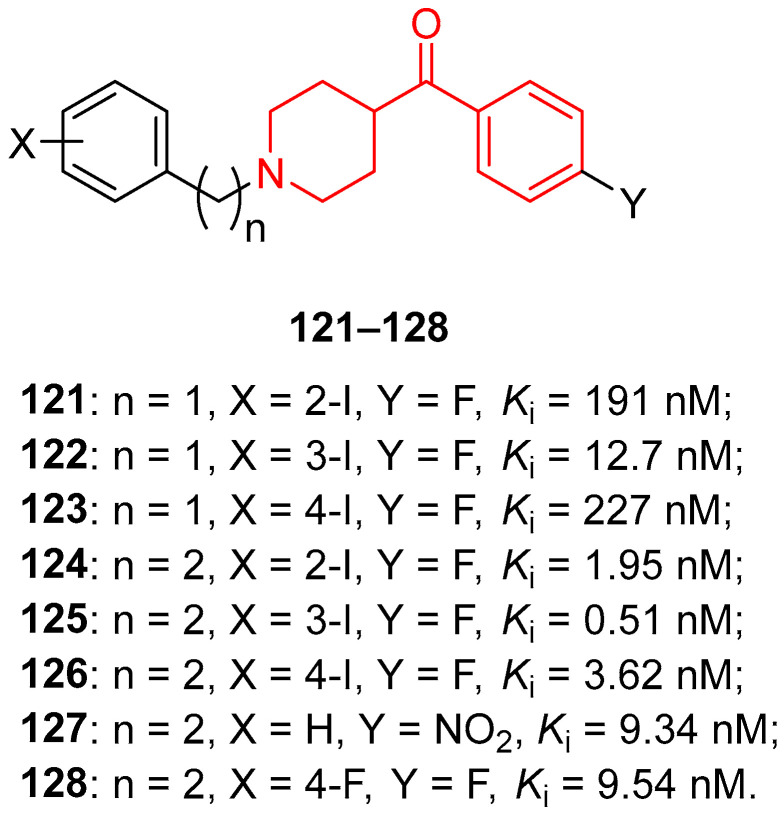
Structures of the benzoylpiperidines **121**–**128** identified by Fu et al. [28] as 5-HT_2A_ ligands for PET or SPECT brain imaging. The benzoylpiperidine fragment is highlighted in red.

In this context, Fu and collaborators developed a new series of radioligands for PET and SPECT imaging based on the same structure as those of **2**, (+)-**54** [28]. They synthesized radioligands **121**–**128** characterized by a benzoylpiperidine moiety (Figure 31).

Neuropharmacological investigations determined the potency of the developed ligands, highlighting that compounds possessing an ethylene bridge (n = 2, compounds **124**–**128**) had a higher affinity for the 5-HT_2A_ receptors (*K*_i_ values ranging from 0.51 to 9.54 nM) compared with their methylene analogues (compounds **121**–**123**). Nevertheless, derivatives **124**–**128** were endowed with a scarce selectivity for 5-HT_2A_ versus 5-HT_2C_ receptors, reducing their potential for use in brain imaging.

Some years later, Blanckaert and colleagues decided to radiolabel compounds **124**–**126**, previously developed by Fu et al. [28] as potential 5-HT_2A_-antagonists, for SPECT brain imaging [29,30,31]. The developed radioligands [^123^I]-**124**–**126** were 5-HT_2A_-antagonists in the nanomolar range (*K*_i_ values of 1.95, 0.51, and 3.62 nM, respectively), and they were properly selective for 5-HT_2A_ receptors (about 40-fold for [^123^I]-**126**, 20-fold for [^123^I]-**124**, and 30-fold for [^123^I]-**125**) over 5-HT_2C_ receptors, as reported by Fu et al. [28]. Noteworthy, the Log*P* values of compounds [^123^I]-**124** and [^123^I]-**125** were good considering that a Log*P* ranging from 2 to 3 is optimal to minimize the risk of a specific binding. Preliminary in vivo evaluation proved an appreciable uptake of the developed tracers in mice’s brains, even for compound [^123^I]-**126** endowed with a Log*P* of 1.43, thus prompting the authors to further investigate in the future the properties of these radioligands in larger animals such as rats and rabbits.

## 4. Conclusions

This comprehensive review, which is focused on bioactive molecules reported in the literature since 2000, demonstrates that the benzoylpiperidine structure is a fragment widely found in compounds of great biological and diagnostic significance and highlights the benzoylpiperidine fragment as an interesting starting point for the design and development of novel therapeutic and diagnostic agents. The class of therapeutic agents is more populated than the diagnostic one, and the majority of them exert neuroprotective effects, even if anticancer and cardioprotective agents represent two other highly populated classes of therapeutics.

The general overview provided on these benzoylpiperidine derivatives could help to better guide the design and lead optimization of several potential bioactive compounds with improved pharmacological, pharmacokinetic, and physicochemical profiles, but also to synthesize more efficient diagnostics.

A specific section is dedicated to the most frequently used synthetic procedures to assemble the benzoylpiperidine fragment. The short and straightforward synthesis of the benzoylpiperidine fragment involves safe and low-cost reagents, thus attracting the scientific community to employ this chemical frame in drug design and development.

Finally, this extensive review could prompt researchers to adopt the divergent synthesis approach by planning efficient synthetic routes to create libraries of novel benzoylpiperidine-based derivatives by using a common intermediate [147,148].

## Figures and Tables

**Table 1:** Structure of atypical antipsychotics developed by the research group of Prof. Masaguer and Prof. Loza (University of Santiago de Compostela) in the 2000s. The benzoylpiperidine fragment is highlighted in red. * IC_50_ values refer to the *minus* isomer.

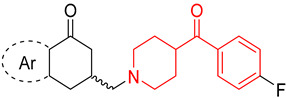		
Ar	Compound	Activity
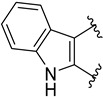	**34**	p*K*_i_ = 8.04 (5-HT_2A_), 6.25 (D_2_)
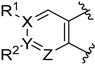	**38**: X = Y = Z = C, R^1^ = R^2^ = H	p*K*_i_ = 8.23 (5-HT_2A_), 6.98 (D_2_) *
	**39**: X = Y = Z = C, R^1^ = R^2^ = OCH_3_	p*K*_i_ = 8.25 (5-HT_2A_), 6.00 (D_2_) *
	**40**: X = Z = N, Y = C, R^2^ = H	*K*_i_ = 32 nM (5-HT_2A_), 160 nM (D_2_)
	**41**: X = C, Y = Z = N, R^1^ = CH_3_	*K*_i_ = 9950 nM (5-HT_2A_), >10,000 nM (D_2_)
	**42**: X = Z = N, Y = C, R^2^ = SCH_3_.	p*K*_i_ = 6.99 (5-HT_2A_)
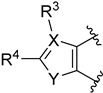	**35**: X = C, Y = O, R^3^ = R^4^ = H	p*K*_i_ = 7.29 (5-HT_2A_), 7.02 (D_2_)
	**47**: X = C, Y = O, R^3^ = COOCH_3_, R^4^ = H	p*K*_i_ = 7.59 (5-HT_2A_), <5 (D_2_)
	**48**: X = C, Y = O, R^3^ = H, R^4^ = Ph	p*K*_i_ = 7.76 (5-HT_2A_), <5 (D_2_)
	**49**: X = C, Y = O, R^3^ = CH_3_, R^4^ = COOCH_2_CH_3_	p*K*_i_ = 7.66 (5-HT_2A_), <5 (D_2_)
	**50**: X = N, Y = O, R^4^ = CH_3_	p*K*_i_ = 6.90 (5-HT_2A_), <5 (D_2_)
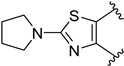	**51**	p*K*_i_ = 6.55 (5-HT_2A_), <5 (D_2_)

## Data Availability

No new data were created or analyzed in this study. Data sharing is not applicable to this article.

## References

[1] Liu Y., Guo L., Duan H., Zhang L., Jiang N., Zhen X., Shen J. (2015). Discovery of 4-Benzoylpiperidine and 3-(Piperidin-4-Yl)Benzo[d]Isoxazole Derivatives as Potential and Selective GlyT1 Inhibitors. RSC Adv..

[2] Yadav V.D., Boshoff H.I., Trifonov L., Roma J.S.O., Ioerger T.R., Barry C.E., Oh S. (2023). Synthesis and Structure–Activity Relationships of a New Class of Oxadiazoles Targeting DprE1 as Antitubercular Agents. ACS Med. Chem. Lett..

[3] Karmacharya U., Chaudhary P., Lim D., Dahal S., Awasthi B.P., Park H.D., Kim J.-A., Jeong B.-S. (2021). Synthesis and Anticancer Evaluation of 6-Azacyclonol-2,4,6-Trimethylpyridin-3-Ol Derivatives: M3 Muscarinic Acetylcholine Receptor-Mediated Anticancer Activity of a Cyclohexyl Derivative in Androgen-Refractory Prostate Cancer. Bioorg. Chem..

[4] Jin J., Zhang K., Dou F., Hao C., Zhang Y., Cao X., Gao L., Xiong J., Liu X., Liu B.-F. (2020). Isoquinolinone Derivatives as Potent CNS Multi-Receptor D2/5-HT1A/5-HT2A/5-HT6/5-HT7 Agents: Synthesis and Pharmacological Evaluation. Eur. J. Med. Chem..

[5] Rolfe A., Yao S., Nguyen T.-V., Omoto K., Colombo F., Virrankoski M., Vaillancourt F.H., Yu L., Cook A., Reynolds D. (2020). Discovery of 2,6-Dimethylpiperazines as Allosteric Inhibitors of CPS1. ACS Med. Chem. Lett..

[6] Fukuda T., Ishiyama T., Katagiri T., Ueda K., Muramatsu S., Hashimoto M., Aki A., Baba D., Watanabe K., Tanaka N. (2018). Discovery of DS42450411 as a Potent Orally Active Hepcidin Production Inhibitor: Design and Optimization of Novel 4-Aminopyrimidine Derivatives. Bioorg. Med. Chem. Lett..

[7] Migliore M., Pontis S., Fuentes de Arriba A.L., Realini N., Torrente E., Armirotti A., Romeo E., Di Martino S., Russo D., Pizzirani D. (2016). Second-Generation Non-Covalent NAAA Inhibitors Are Protective in a Model of Multiple Sclerosis. Angew. Chem. Int. Ed..

[8] Jones A.M. (2017). Privileged Structures and Motifs (Synthetic and Natural Scaffolds). Comprehensive Medicinal Chemistry III.

[9] Duncan R.L., Helsley G.C., Welstead W.J., DaVanzo J.P., Funderburk W.H., Lunsford C.D. (1970). Aroylpiperidines and Pyrrolidines. New Class of Potent Central Nervous System Depressants. J. Med. Chem..

[10] Ismaiel A.M., Arruda K., Teitler M., Glennon R.A. (1995). Ketanserin Analogs: The Effect of Structural Modification on 5-HT2 Serotonin Receptor Binding. J. Med. Chem..

[11] Masaguer C. (2000). Butyrophenone Analogues in the Carbazole Series as Potential Atypical Antipsychotics: Synthesis and Determination of Affinities at D2, 5-HT2A, 5-HT2B and 5-HT2C Receptors. Eur. J. Med. Chem..

[12] Herndon J.L., Ismaiel A., Ingher S.P., Teitler M., Glennon R.A. (1992). Ketanserin Analogs: Structure-Affinity Relationships for 5-HT2 and 5-HT1C Serotonin Receptor Binding. J. Med. Chem..

[13] Tuccinardi T., Granchi C., Rizzolio F., Caligiuri I., Battistello V., Toffoli G., Minutolo F., Macchia M., Martinelli A. (2014). Identification and Characterization of a New Reversible MAGL Inhibitor. Bioorg. Med. Chem..

[14] Carro L., Raviña E., Domínguez E., Brea J., Loza M.I., Masaguer C.F. (2009). Synthesis and Binding Affinity of Potential Atypical Antipsychotics with the Tetrahydroquinazolinone Motif. Bioorg. Med. Chem. Lett..

[15] Kramer V., Herth M.M., Santini M.A., Palner M., Knudsen G.M., Rösch F. (2010). Research Letter: Structural Combination of Established 5-HT2A Receptor Ligands: New Aspects of the Binding Mode. Chem. Biol. Drug Des..

[16] Barceló M., Raviña E., Varela M.J., Brea J., Loza M.I., Masaguer C.F. (2011). Potential Atypical Antipsychotics: Synthesis, Binding Affinity and SAR of New Heterocyclic Bioisosteric Butyrophenone Analogues as Multitarget Ligands. Medchemcomm.

[17] Carro L., Torrado M., Raviña E., Masaguer C.F., Lage S., Brea J., Loza M.I. (2014). Synthesis and Biological Evaluation of a Series of Aminoalkyl-Tetralones and Tetralols as Dual Dopamine/Serotonin Ligands. Eur. J. Med. Chem..

[18] Wang W., Cui J., Lu X., Padakanti P.K., Xu J., Parsons S.M., Luedtke R.R., Rath N.P., Tu Z. (2011). Synthesis and In Vitro Biological Evaluation of Carbonyl Group-Containing Analogues for Σ1 Receptors. J. Med. Chem..

[19] Ikome H.N., Ntie-Kang F., Ngemenya M.N., Tu Z., Mach R.H., Efange S.M.N. (2016). 4-Aroylpiperidines and 4-(α-Hydroxyphenyl)Piperidines as Selective Sigma-1 Receptor Ligands: Synthesis, Preliminary Pharmacological Evaluation and Computational Studies. Chem. Cent. J..

[20] Gupta M., Ojha M., Yadav D., Pant S., Yadav R. (2020). Novel Benzylated (Pyrrolidin-2-One)/(Imidazolidin-2-One) Derivatives as Potential Anti-Alzheimer’s Agents: Synthesis and Pharmacological Investigations. ACS Chem. Neurosci..

[21] Zheng Y., Müller J., Kunz S., Siderius M., Maes L., Caljon G., Müller N., Hemphill A., Sterk G.J., Leurs R. (2022). 3-Nitroimidazo[1,2-b]Pyridazine as a Novel Scaffold for Antiparasitics with Sub-Nanomolar Anti-Giardia Lamblia Activity. Int. J. Parasitol. Drugs Drug Resist..

[22] Jenkins Y., Sun T.-Q., Markovtsov V., Foretz M., Li W., Nguyen H., Li Y., Pan A., Uy G., Gross L. (2013). AMPK Activation through Mitochondrial Regulation Results in Increased Substrate Oxidation and Improved Metabolic Parameters in Models of Diabetes. PLoS ONE.

[23] Louvel J., Carvalho J.F.S., Yu Z., Soethoudt M., Lenselink E.B., Klaasse E., Brussee J., IJzerman A.P. (2013). Removal of Human Ether-à-Go-Go Related Gene (HERG) K + Channel Affinity through Rigidity: A Case of Clofilium Analogues. J. Med. Chem..

[24] Granchi C., Rizzolio F., Palazzolo S., Carmignani S., Macchia M., Saccomanni G., Manera C., Martinelli A., Minutolo F., Tuccinardi T. (2016). Structural Optimization of 4-Chlorobenzoylpiperidine Derivatives for the Development of Potent, Reversible, and Selective Monoacylglycerol Lipase (MAGL) Inhibitors. J. Med. Chem..

[25] Li Q., Chai L., Dong G., Zhang X., Du L. (2021). NBD-Based Environment-Sensitive Fluorescent Probes for the Human Ether-a-Go-Go–Related Gene Potassium Channel. Front. Mol. Biosci..

[26] Liebeschuetz J.W., Jones S.D., Morgan P.J., Murray C.W., Rimmer A.D., Roscoe J.M.E., Waszkowycz B., Welsh P.M., Wylie W.A., Young S.C. (2002). PRO_SELECT: Combining Structure-Based Drug Design and Array-Based Chemistry for Rapid Lead Discovery. 2. The Development of a Series of Highly Potent and Selective Factor Xa Inhibitors. J. Med. Chem..

[27] Barthel C., Sorger D., Deuther-Conrad W., Scheunemann M., Schweiger S., Jäckel P., Roghani A., Steinbach J., Schüürmann G., Sabri O. (2015). New Systematically Modified Vesamicol Analogs and Their Affinity and Selectivity for the Vesicular Acetylcholine Transporter—A Critical Examination of the Lead Structure. Eur. J. Med. Chem..

[28] Fu X., Tan P.-Z., Kula N.S., Baldessarini R., Tamagnan G., Innis R.B., Baldwin R.M. (2002). Synthesis, Receptor Potency, and Selectivity of Halogenated Diphenylpiperidines as Serotonin 5-HT 2A Ligands for PET or SPECT Brain Imaging. J. Med. Chem..

[29] Blanckaert P., Vandecapelle M., Staelens L., Burvenich I., Dierckx R.A., Slegers G. (2004). Synthesis, Radiosynthesis and Preliminaryin Vivo Evaluation of[123I]-(4-Fluorophenyl){1-[2-(2-Iodophenyl)Ethyl]Piperidin-4-Yl}methanone, a Potential 5-HT2A-Antagonist for SPECT Brain Imaging. J. Label. Compd. Radiopharm..

[30] Blanckaert P., Burvenich I., Staelens L., Dierckx R.A., Slegers G. (2005). Synthesis, Radiosynthesis and in Vivo Evaluation in Mice of [123I]-(4-Fluorophenyl) {1-[2-(4-Iodophenyl)Ethyl]Piperidin-4-Yl}methanone for Visualization of the 5-HT2A Receptor with SPECT. Appl. Radiat. Isot..

[31] Blanckaert P., Burvenich I., Devos F., Slegers G. (2007). Synthesis Andin Vivo Evaluation in Mice of [123I]-(4-Fluorophenyl)[1-(3-Iodophenethyl)Piperidin-4-Yl]Methanone as a Potential SPECT-Tracer for the Serotonin 5-HT2A Receptor. J. Label. Compd. Radiopharm..

[32] Shultz M.D., Cheung A.K., Kirby C.A., Firestone B., Fan J., Chen C.H.-T., Chen Z., Chin D.N., DiPietro L., Fazal A. (2013). Identification of NVP-TNKS656: The Use of Structure–Efficiency Relationships to Generate a Highly Potent, Selective, and Orally Active Tankyrase Inhibitor. J. Med. Chem..

[33] Comoy C., Guérin V., Pfeiffer B., Rettori M.-C., Renard P., Guillaumet G. (2000). Substituted 3-Amino and/or 3-Aminomethyl-3,4-Dihydro-2H-1-Benzopyrans: Synthesis and Biological Activity. Bioorg. Med. Chem..

[34] Raviña E., Casariego I., Masaguer C.F., Fontenla J.A., Montenegro G.Y., Rivas M.E., Loza M.I., Enguix M.J., Villazon M., Cadavid M.I. (2000). Conformationally Constrained Butyrophenones with Affinity for Dopamine (D_1_, D_2_, D_4_) and Serotonin (5-HT_2A_, 5-HT_2B_, 5-HT_2C_) Receptors:  Synthesis of Aminomethylbenzo[*b*]Furanones and Their Evaluation as Antipsychotics. J. Med. Chem..

[35] Caro Y., Torrado M., Masaguer C.F., Raviña E., Padín F., Brea J., Loza M.I. (2004). Chemoenzymatic Synthesis and Binding Affinity of Novel (R)- and (S)-3-Aminomethyl-1-Tetralones, Potential Atypical Antipsychotics. Bioorg. Med. Chem. Lett..

[36] Alvarado M., Barceló M., Carro L., Masaguer C.F., Raviña E. (2006). Synthesis and Biological Evaluation of New Quinazoline and Cinnoline Derivatives as Potential Atypical Antipsychotics. Chem. Biodivers..

[37] Barceló M., Raviña E., Masaguer C.F., Domínguez E., Areias F.M., Brea J., Loza M.I. (2007). Synthesis and Binding Affinity of New Pyrazole and Isoxazole Derivatives as Potential Atypical Antipsychotics. Bioorg. Med. Chem. Lett..

[38] Aranda R., Villalba K., Raviña E., Masaguer C.F., Brea J., Areias F., Domínguez E., Selent J., López L., Sanz F. (2008). Synthesis, Binding Affinity, and Molecular Docking Analysis of New Benzofuranone Derivatives as Potential Antipsychotics. J. Med. Chem..

[39] Granchi C., Lapillo M., Glasmacher S., Bononi G., Licari C., Poli G., el Boustani M., Caligiuri I., Rizzolio F., Gertsch J. (2019). Optimization of a Benzoylpiperidine Class Identifies a Highly Potent and Selective Reversible Monoacylglycerol Lipase (MAGL) Inhibitor. J. Med. Chem..

[40] Granchi C., Bononi G., Ferrisi R., Gori E., Mantini G., Glasmacher S., Poli G., Palazzolo S., Caligiuri I., Rizzolio F. (2021). Design, Synthesis and Biological Evaluation of Second-Generation Benzoylpiperidine Derivatives as Reversible Monoacylglycerol Lipase (MAGL) Inhibitors. Eur. J. Med. Chem..

[41] Bononi G., Tonarini G., Poli G., Barravecchia I., Caligiuri I., Macchia M., Rizzolio F., Demontis G.C., Minutolo F., Granchi C. (2021). Monoacylglycerol Lipase (MAGL) Inhibitors Based on a Diphenylsulfide-Benzoylpiperidine Scaffold. Eur. J. Med. Chem..

[42] Zhong Y., Gao Y., Xu Y., Qi C., Wu B. (2020). Synthesis of Novel Aryloxyethylamine Derivatives and Evaluation of Their In Vitro and In Vivo Neuroprotective Activities. Chem. Biodivers..

[43] Li D., Gao N., Zhu N., Lin Y., Li Y., Chen M., You X., Lu Y., Wan K., Jiang J.-D. (2015). Discovery of the Disubstituted Oxazole Analogues as a Novel Class Anti-Tuberculotic Agents against MDR- and XDR-MTB. Bioorg. Med. Chem. Lett..

[44] Vilums M., Overman J., Klaasse E., Scheel O., Brussee J., IJzerman A.P. (2012). Understanding of Molecular Substructures That Contribute to HERG K + Channel Blockade: Synthesis and Biological Evaluation of E-4031 Analogues. ChemMedChem.

[45] Nahm S., Weinreb S.M. (1981). N-Methoxy-n-Methylamides as Effective Acylating Agents. Tetrahedron Lett..

[46] Shashack M.J., Cunningham K.A., Seitz P.K., McGinnis A., Smith T.D., Watson C.S., Gilbertson S.R. (2011). Synthesis and Evaluation of Dimeric Derivatives of 5-HT_2A_ Receptor (5-HT_2A_R) Antagonist M-100907. ACS Chem. Neurosci..

[47] Soto C.A., Shashack M.J., Fox R.G., Bubar M.J., Rice K.C., Watson C.S., Cunningham K.A., Gilbertson S.R., Anastasio N.C. (2018). Novel Bivalent 5-HT 2A Receptor Antagonists Exhibit High Affinity and Potency In Vitro and Efficacy In Vivo. ACS Chem. Neurosci..

[48] Gilbertson S.R., Chen Y.-C., Soto C.A., Yang Y., Rice K.C., Cunningham K.A., Anastasio N.C. (2018). Synthesis and Activity of Functionalizable Derivatives of the Serotonin (5-HT) 5-HT_2A_ Receptor (5-HT_2A_R) Antagonist M100907. Bioorg. Med. Chem. Lett..

[49] Poulie C.B.M., Liu N., Jensen A.A., Bunch L. (2020). Design, Synthesis, and Pharmacological Characterization of Heterobivalent Ligands for the Putative 5-HT_2A_/MGlu_2_ Receptor Complex. J. Med. Chem..

[50] Deau E., Robin E., Voinea R., Percina N., Satała G., Finaru A.-L., Chartier A., Tamagnan G., Alagille D., Bojarski A.J. (2015). Rational Design, Pharmacomodulation, and Synthesis of Dual 5-Hydroxytryptamine 7 (5-HT_7_)/5-Hydroxytryptamine 2A (5-HT_2A_) Receptor Antagonists and Evaluation by [^18^F]-PET Imaging in a Primate Brain. J. Med. Chem..

[51] Tu Z., Efange S.M.N., Xu J., Li S., Jones L.A., Parsons S.M., Mach R.H. (2009). Synthesis and In Vitro and In Vivo Evaluation of ^18^F-Labeled Positron Emission Tomography (PET) Ligands for Imaging the Vesicular Acetylcholine Transporter. J. Med. Chem..

[52] Yue X., Luo Z., Liu H., Kaneshige K., Parsons S.M., Perlmutter J.S., Tu Z. (2018). Radiosynthesis and Evaluation of a Fluorine-18 Labeled Radioligand Targeting Vesicular Acetylcholine Transporter. Bioorg. Med. Chem. Lett..

[53] Efange S.M.N., Khare A.B., von Hohenberg K., Mach R.H., Parsons S.M., Tu Z. (2010). Synthesis and *in Vitro* Biological Evaluation of Carbonyl Group-Containing Inhibitors of Vesicular Acetylcholine Transporter. J. Med. Chem..

[54] Tu Z., Wang W., Cui J., Zhang X., Lu X., Xu J., Parsons S.M. (2012). Synthesis and Evaluation of In Vitro Bioactivity for Vesicular Acetylcholine Transporter Inhibitors Containing Two Carbonyl Groups. Bioorg. Med. Chem..

[55] Li J., Zhang X., Zhang Z., Padakanti P.K., Jin H., Cui J., Li A., Zeng D., Rath N.P., Flores H. (2013). Heteroaromatic and Aniline Derivatives of Piperidines As Potent Ligands for Vesicular Acetylcholine Transporter. J. Med. Chem..

[56] Liu H., Jin H., Li J., Zhang X., Kaneshige K., Parsons S.M., Perlmutter J.S., Tu Z. (2015). In Vitro and Ex Vivo Characterization of (−)-TZ659 as a Ligand for Imaging the Vesicular Acetylcholine Transporter. Eur. J. Pharmacol..

[57] Padakanti P.K., Zhang X., Jin H., Cui J., Wang R., Li J., Flores H.P., Parsons S.M., Perlmutter J.S., Tu Z. (2014). In Vitro and In Vivo Characterization of Two C-11-Labeled PET Tracers for Vesicular Acetylcholine Transporter. Mol. Imaging Biol..

[58] Padakanti P.K., Zhang X., Li J., Parsons S.M., Perlmutter J.S., Tu Z. (2014). Syntheses and Radiosyntheses of Two Carbon-11 Labeled Potent and Selective Radioligands for Imaging Vesicular Acetylcholine Transporter. Mol. Imaging Biol..

[59] Tu Z., Zhang X., Jin H., Yue X., Padakanti P.K., Yu L., Liu H., Flores H.P., Kaneshige K., Parsons S.M. (2015). Synthesis and Biological Characterization of a Promising F-18 PET Tracer for Vesicular Acetylcholine Transporter. Bioorg. Med. Chem..

[60] Yue X., Jin H., Liu H., Luo Z., Zhang X., Kaneshige K., Flores H.P., Perlmutter J.S., Parsons S.M., Tu Z. (2017). Synthesis, Resolution, and in Vitro Evaluation of Three Vesicular Acetylcholine Transporter Ligands and Evaluation of the Lead Fluorine-18 Radioligand in a Nonhuman Primate. Org. Biomol. Chem..

[61] Uto Y., Ogata T., Kiyotsuka Y., Ueno Y., Miyazawa Y., Kurata H., Deguchi T., Watanabe N., Konishi M., Okuyama R. (2010). Novel Benzoylpiperidine-Based Stearoyl-CoA Desaturase-1 Inhibitors: Identification of 6-[4-(2-Methylbenzoyl)Piperidin-1-Yl]Pyridazine-3-Carboxylic Acid (2-Hydroxy-2-Pyridin-3-Ylethyl)Amide and Its Plasma Triglyceride-Lowering Effects in Zucker Fatty Rats. Bioorg. Med. Chem. Lett..

[62] Sander K., Galante E., Gendron T., Yiannaki E., Patel N., Kalber T.L., Badar A., Robson M., Johnson S.P., Bauer F. (2015). Development of Fluorine-18 Labeled Metabolically Activated Tracers for Imaging of Drug Efflux Transporters with Positron Emission Tomography. J. Med. Chem..

[63] Mulvihill M.M., Nomura D.K. (2013). Therapeutic Potential of Monoacylglycerol Lipase Inhibitors. Life Sci..

[64] Schlosburg J.E., Blankman J.L., Long J.Z., Nomura D.K., Pan B., Kinsey S.G., Nguyen P.T., Ramesh D., Booker L., Burston J.J. (2010). Chronic Monoacylglycerol Lipase Blockade Causes Functional Antagonism of the Endocannabinoid System. Nat. Neurosci..

[65] Haikarainen T., Krauss S., Lehtio L. (2014). Tankyrases: Structure, Function and Therapeutic Implications in Cancer. Curr. Pharm. Des..

[66] Huang S.-M.A., Mishina Y.M., Liu S., Cheung A., Stegmeier F., Michaud G.A., Charlat O., Wiellette E., Zhang Y., Wiessner S. (2009). Tankyrase Inhibition Stabilizes Axin and Antagonizes Wnt Signalling. Nature.

[67] Polakis P. (2007). The Many Ways of Wnt in Cancer. Curr. Opin. Genet. Dev..

[68] Sharma L., Lu J., Bai Y. (2009). Mitochondrial Respiratory Complex I: Structure, Function and Implication in Human Diseases. Curr. Med. Chem..

[69] Hirst J. (2013). Mitochondrial Complex I. Annu. Rev. Biochem..

[70] Pollak M. (2013). Targeting Oxidative Phosphorylation: Why, When, and How. Cancer Cell.

[71] Huang Y., Sun G., Wang P., Shi R., Zhang Y., Wen X., Sun H., Chen C. (2018). Synthesis and Biological Evaluation of Complex I Inhibitor R419 and Its Derivatives as Anticancer Agents in HepG2 Cells. Bioorg. Med. Chem. Lett..

[72] Lin J., Liu W., Guan J., Cui J., Shi R., Wang L., Chen D., Liu Y. (2023). Latest Updates on the Serotonergic System in Depression and Anxiety. Front. Synaptic Neurosci..

[73] Brasso C., Colli G., Sgro R., Bellino S., Bozzatello P., Montemagni C., Villari V., Rocca P. (2023). Efficacy of Serotonin and Dopamine Activity Modulators in the Treatment of Negative Symptoms in Schizophrenia: A Rapid Review. Biomedicines.

[74] Leibowitz S.F. (1990). The Role of Serotonin in Eating Disorders. Drugs.

[75] Sinopoli V.M., Burton C.L., Kronenberg S., Arnold P.D. (2017). A Review of the Role of Serotonin System Genes in Obsessive-Compulsive Disorder. Neurosci. Biobehav. Rev..

[76] Johnson K.W., Phebus L.A., Cohen M.L. (1998). Serotonin in Migraine: Theories, Animal Models and Emerging Therapies. Progress in Drug Research.

[77] Graeff F.G. (2017). Translational Approach to the Pathophysiology of Panic Disorder: Focus on Serotonin and Endogenous Opioids. Neurosci. Biobehav. Rev..

[78] McCorvy J.D., Roth B.L. (2015). Structure and Function of Serotonin G Protein-Coupled Receptors. Pharmacol. Ther..

[79] Staroń J., Bugno R., Hogendorf A.S., Bojarski A.J. (2018). 5-HT1A Receptor Ligands and Their Therapeutic Applications: Review of New Patents. Expert Opin. Ther. Pat..

[80] Nagatomo T., Rashid M., Abul Muntasir H., Komiyama T. (2004). Functions of 5-HT2A Receptor and Its Antagonists in the Cardiovascular System. Pharmacol. Ther..

[81] van Rossum J.M. (1966). The Significance of Dopamine-Receptor Blockade for the Mechanism of Action of Neuroleptic Drugs. Arch. Int. Pharmacodyn. Ther..

[82] Brea J., Rodrigo J., Carrieri A., Sanz F., Cadavid M.I., Enguix M.J., Villazón M., Mengod G., Caro Y., Masaguer C.F. (2002). New Serotonin 5-HT_2A_, 5-HT_2B_, and 5-HT_2C_ Receptor Antagonists: Synthesis, Pharmacology, 3D-QSAR, and Molecular Modeling of (Aminoalkyl)Benzo and Heterocycloalkanones. J. Med. Chem..

[83] Fontenla J.A., Osuna J., Rosa E., Castro M.E., G.-Ferreiro T., Loza-Garcia I., Calleja J.M., Sanz F., Rodriguez J. (1994). Synthesis and Atypical Antipsychotic Profile of Some 2-(2-Piperidinoethyl)Benzocycloalkanones as Analogs of Butyrophenone. J. Med. Chem..

[84] Masaguer C.F., Casariego I., Ravina E. (1999). Conformationally Restricted Butyrophenones with Mixed Dopaminergic (D_2_) and Serotoninergic (5-HT_2A_) Affinities. Synthesis of 5-Aminoethyl and 6-Aminomethyl-4-Oxotetrahydroindoles as Potential Atypical Antipsychotics. Chem. Pharm. Bull..

[85] Herrick-Davis K., Grinde E., Harrigan T.J., Mazurkiewicz J.E. (2005). Inhibition of Serotonin 5-Hydroxytryptamine2C Receptor Function through Heterodimerization. J. Biol. Chem..

[86] Pellissier L.P., Barthet G., Gaven F., Cassier E., Trinquet E., Pin J.-P., Marin P., Dumuis A., Bockaert J., Banères J.-L. (2011). G Protein Activation by Serotonin Type 4 Receptor Dimers. J. Biol. Chem..

[87] Barnes N.M., Sharp T. (1999). A Review of Central 5-HT Receptors and Their Function. Neuropharmacology.

[88] Hedlund P.B. (2009). The 5-HT7 Receptor and Disorders of the Nervous System: An Overview. Psychopharmacology.

[89] De Filippis B., Nativio P., Fabbri A., Ricceri L., Adriani W., Lacivita E., Leopoldo M., Passarelli F., Fuso A., Laviola G. (2014). Pharmacological Stimulation of the Brain Serotonin Receptor 7 as a Novel Therapeutic Approach for Rett Syndrome. Neuropsychopharmacology.

[90] Yasuhara A., Chaki S. (2010). Metabotropic Glutamate Receptors: Potential Drug Targets for Psychiatric Disorders. Open Med. Chem. J..

[91] González-Maeso J., Ang R.L., Yuen T., Chan P., Weisstaub N.V., López-Giménez J.F., Zhou M., Okawa Y., Callado L.F., Milligan G. (2008). Identification of a Serotonin/Glutamate Receptor Complex Implicated in Psychosis. Nature.

[92] Baki L., Fribourg M., Younkin J., Eltit J.M., Moreno J.L., Park G., Vysotskaya Z., Narahari A., Sealfon S.C., Gonzalez-Maeso J. (2016). Cross-Signaling in Metabotropic Glutamate 2 and Serotonin 2A Receptor Heteromers in Mammalian Cells. Pflügers Arch.—Eur. J. Physiol..

[93] Moreno J.L., Miranda-Azpiazu P., García-Bea A., Younkin J., Cui M., Kozlenkov A., Ben-Ezra A., Voloudakis G., Fakira A.K., Baki L. (2016). Allosteric Signaling through an MGlu2 and 5-HT_2A_ Heteromeric Receptor Complex and Its Potential Contribution to Schizophrenia. Sci. Signal..

[94] Danysz W., Parsons C.G. (1998). Glycine and N-Methyl-D-Aspartate Receptors: Physiological Significance and Possible Therapeutic Applications. Pharmacol. Rev..

[95] Kantrowitz J., Javitt D.C. (2012). Glutamatergic Transmission in Schizophrenia. Curr. Opin. Psychiatry.

[96] Marino M.J., Knutsen L.J.S., Williams M. (2008). Emerging Opportunities for Antipsychotic Drug Discovery in the Postgenomic Era. J. Med. Chem..

[97] Sur C., Kinney G. (2007). Glycine Transporter 1 Inhibitors and Modulation of NMDA Receptor-Mediated Excitatory Neurotransmission. Curr. Drug Targets.

[98] Raiteri L., Raiteri M. (2010). Functional ‘Glial’ GLYT1 Glycine Transporters Expressed in Neurons. J. Neurochem..

[99] Pinard E., Alanine A., Alberati D., Bender M., Borroni E., Bourdeaux P., Brom V., Burner S., Fischer H., Hainzl D. (2010). Selective GlyT1 Inhibitors: Discovery of [4-(3-Fluoro-5-Trifluoromethylpyridin-2-Yl)Piperazin-1-Yl][5-Methanesulfonyl-2-((S)-2,2,2-Trifluoro-1-Methylethoxy)Phenyl]Methanone (RG1678), a Promising Novel Medicine To Treat Schizophrenia. J. Med. Chem..

[100] Shen J., Zhen X., Duan H., Guo L., Zhang L., Zhu L. (2014). Piperidine Compounds, and Preparation Method, Pharmaceutical Compositions and Use Thereof. U.S. Patent.

[101] Bourrie B., Bribes E., Derocq J.-M., Vidal H., Casellas P. (2004). Sigma Receptor Ligands: Applications in Inflammation and Oncology. Curr. Opin. Investig. Drugs.

[102] Bowen W.D. (2000). Sigma Receptors: Recent Advances and New Clinical Potentials. Pharm. Acta Helv..

[103] Ogawa K., Shiba K., Akhter N., Yoshimoto M., Washiyama K., Kinuya S., Kawai K., Mori H. (2009). Evaluation of Radioiodinated Vesamicol Analogs for Sigma Receptor Imaging in Tumor and Radionuclide Receptor Therapy. Cancer Sci..

[104] John C.S., Bowen W.D., Varma V.M., McAfee J.G., Moody T.W. (1995). Sigma Receptors Are Expressed in Human Non-Small Cell Lung Carcinoma. Life Sci..

[105] Vilner B.J., John C.S., Bowen W.D. (1995). Sigma-1 and Sigma-2 Receptors Are Expressed in a Wide Variety of Human and Rodent Tumor Cell Lines. Cancer Res..

[106] John C.S., Gulden M.E., Li J., Bowen W.D., McAfee J.G., Thakur M.L. (1998). Synthesis, In Vitro Binding, and Tissue Distribution of Radioiodinated 2-[^125^I]N-(N-Benzylpiperidin-4-Yl)-2-Iodo Benzamide, 2-[^125^I]BP: A Potential σ Receptor Marker for Human Prostate Tumors. Nucl. Med. Biol..

[107] Quaglia W., Giannella M., Piergentili A., Pigini M., Brasili L., Di Toro R., Rossetti L., Spampinato S., Melchiorre C. (1998). 1′-Benzyl-3,4-Dihydrospiro[2H-1-Benzothiopyran-2,4′-Piperidine] (Spipethiane), a Potent and Highly Selective σ 1 Ligand. J. Med. Chem..

[108] Terry A.V., Buccafusco J.J. (2003). The Cholinergic Hypothesis of Age and Alzheimer’s Disease-Related Cognitive Deficits: Recent Challenges and Their Implications for Novel Drug Development. J. Pharmacol. Exp. Ther..

[109] Gupta M., Kumar A., Prasun C., Nair M.S., Kini S.G., Yadav D., Nain S. (2023). Design, Synthesis, Extra-Precision Docking, and Molecular Dynamics Simulation Studies of Pyrrolidin-2-One Derivatives as Potential Acetylcholinesterase Inhibitors. J. Biomol. Struct. Dyn..

[110] Dye C., Williams B.G. (2010). The Population Dynamics and Control of Tuberculosis. Science.

[111] Elzinga G., Raviglione M.C., Maher D. (2004). Scale up: Meeting Targets in Global Tuberculosis Control. Lancet.

[112] Maitre T., Baulard A., Aubry A., Veziris N. (2023). Optimizing the Use of Current Antituberculosis Drugs to Overcome Drug Resistance in Mycobacterium Tuberculosis. Infect. Dis. Now.

[113] Thompson R.C.A. (2000). Giardiasis as a Re-Emerging Infectious Disease and Its Zoonotic Potential. Int. J. Parasitol..

[114] el-Sayad M.H., Lotfy W.M., El-Kholy S.M., Yehia M.A.H. (2002). Efficacy of Praziquantel against Giardia Lamblia in Rats: Parasitological, Pathological and Therapeutic Study. J. Egypt. Soc. Parasitol..

[115] Rosenblatt J.E. (1999). Antiparasitic Agents. Mayo Clin. Proc..

[116] Vanelle P., Maldonado J., Gasquet M., Delmas F., Timon-David P., Jentzer O., Crozet M.P. (2011). Studies on Antiparasitic Agents: Effect of the Lactam Nucleus Substitution in the 2-Position on the In-Vitro Activity of New 5-Nitroimidazoles. J. Pharm. Pharmacol..

[117] Upcroft J.A., Campbell R.W., Benakli K., Upcroft P., Vanelle P. (1999). Efficacy of New 5-Nitroimidazoles against Metronidazole-Susceptible and -Resistant *Giardia*, *Trichomonas*, and *Entamoeba* Spp. Antimicrob. Agents Chemother..

[118] Tomcufcik A., Izzo P., Fabio P. (1974). 6-Substituted 3-nitroimidazo[1,2-b]pyridazines and Method of Preparing Same. U.S. Patent.

[119] Dobrzyn A., Ntambi J.M. (2005). Stearoyl-CoA Desaturase as a New Drug Target for Obesity Treatment. Obes. Rev..

[120] Ntambi J.M., Miyazaki M. (2003). Recent Insights into Stearoyl-CoA Desaturase-1. Curr. Opin. Lipidol..

[121] Wang J., Yu L., Schmidt R.E., Su C., Huang X., Gould K., Cao G. (2005). Characterization of HSCD5, a Novel Human Stearoyl-CoA Desaturase Unique to Primates. Biochem. Biophys. Res. Commun..

[122] Zhang L., Ge L., Parimoo S., Stenn K., Prouty S.M. (1999). Human Stearoyl-CoA Desaturase: Alternative Transcripts Generated from a Single Gene by Usage of Tandem Polyadenylation Sites. Biochem. J..

[123] Miyazaki M., Kim Y.-C., Gray-Keller M.P., Attie A.D., Ntambi J.M. (2000). The Biosynthesis of Hepatic Cholesterol Esters and Triglycerides Is Impaired in Mice with a Disruption of the Gene for Stearoyl-CoA Desaturase 1. J. Biol. Chem..

[124] Flowers M.T., Ntambi J.M. (2008). Role of Stearoyl-Coenzyme A Desaturase in Regulating Lipid Metabolism. Curr. Opin. Lipidol..

[125] Sivitz W.I., Yorek M.A. (2010). Mitochondrial Dysfunction in Diabetes: From Molecular Mechanisms to Functional Significance and Therapeutic Opportunities. Antioxid. Redox Signal..

[126] Zhang B.B., Zhou G., Li C. (2009). AMPK: An Emerging Drug Target for Diabetes and the Metabolic Syndrome. Cell Metab..

[127] Hitoshi Y., Jenkins Y., Markovtsov V., Kinsella T., Sun T. (2015). Methods for Using and Biomarkers for AMPK-Activating Compounds. U.S. Patent.

[128] Shaw S.J., Goff D.A., Carroll D.C., Singh R., Sweeny D.J., Park G., Jenkins Y., Markovtsov V., Sun T.-Q., Issakani S.D. (2022). Structure Activity Relationships Leading to the Identification of the Indirect Activator of AMPK, R419. Bioorg. Med. Chem..

[129] Shaw S.J., Goff D.A., Boralsky L.A., Singh R., Sweeny D.J., Park G., Sun T.-Q., Jenkins Y., Markovtsov V., Issakani S.D. (2023). Optimization of Pharmacokinetic and In Vitro Safety Profile of a Series of Pyridine Diamide Indirect AMPK Activators. J. Med. Chem..

[130] Raschi E., Vasina V., Poluzzi E., De Ponti F. (2008). The HERG K+ Channel: Target and Antitarget Strategies in Drug Development. Pharmacol. Res..

[131] Yu Z., van Veldhoven J.P.D., Louvel J., ’t Hart I.M.E., Rook M.B., van der Heyden M.A.G., Heitman L.H., IJzerman A.P. (2015). Structure–Affinity Relationships (SARs) and Structure–Kinetics Relationships (SKRs) of K v 11.1 Blockers. J. Med. Chem..

[132] Jehle J., Schweizer P.A., Katus H.A., Thomas D. (2011). Novel Roles for HERG K+ Channels in Cell Proliferation and Apoptosis. Cell Death Dis..

[133] Gross P.L., Weitz J.I. (2009). New Antithrombotic Drugs. Clin. Pharmacol. Ther..

[134] Pinto D.J.P., Smallheer J.M., Cheney D.L., Knabb R.M., Wexler R.R. (2010). Factor Xa Inhibitors: Next-Generation Antithrombotic Agents. J. Med. Chem..

[135] Jones S.D., Liebeschuetz J.W., Morgan P.J., Murray C.W., Rimmer A.D., Roscoe J.M., Waszkowycz B., Welsh P.M., Wylie W.A., Young S.C. (2001). The Design of Phenylglycine Containing Benzamidine Carboxamides as Potent and Selective Inhibitors of Factor Xa. Bioorg. Med. Chem. Lett..

[136] Hieble J.P. (2007). Recent Advances in Identification and Characterization of β-Adrenoceptor Agonists and Antagonists. Curr. Top. Med. Chem..

[137] Kolb P., Rosenbaum D.M., Irwin J.J., Fung J.J., Kobilka B.K., Shoichet B.K. (2009). Structure-Based Discovery of β_2_-Adrenergic Receptor Ligands. Proc. Natl. Acad. Sci. USA.

[138] Rouget C., Breuiller-Fouché M., Mercier F.J., Leroy M.J., Loustalot C., Naline E., Frydman R., Croci T., Morcillo E.J., Advenier C. (2004). The Human Near-term Myometrial β_3_-adrenoceptor but Not the β_2_-adrenoceptor Is Resistant to Desensitisation after Sustained Agonist Stimulation. Br. J. Pharmacol..

[139] Inoue Y., Yoshizato T., Kawarabayashi T. (2009). Investigation of β_2_-adrenoceptor Subtype Selectivity and Organ Specificity for Bedoradrine (KUR-1246), a Novel Tocolytic Beta-adrenergic Receptor Stimulant. J. Obstet. Gynaecol. Res..

[140] Doggrell S.A. (2004). Recent Pharmacological Advances in the Treatment of Preterm Membrane Rupture, Labour and Delivery. Expert Opin. Pharmacother..

[141] Tasler S., Baumgartner R., Aschenbrenner A., Ammendola A., Wolf K., Wieber T., Schachtner J., Blisse M., Quotschalla U., Ney P. (2010). A VHTS Approach for the Identification of β-Adrenoceptor Ligands. Bioorg. Med. Chem. Lett..

[142] Bullock R., Touchon J., Bergman H., Gambina G., He Y., Rapatz G., Nagel J., Lane R. (2005). Rivastigmine and Donepezil Treatment in Moderate to Moderately-Severe Alzheimer’s Disease over a 2-Year Period. Curr. Med. Res. Opin..

[143] Bravo D.T., Kolmakova N.G., Parsons S.M. (2004). Choline Is Transported by Vesicular Acetylcholine Transporter. J. Neurochem..

[144] Lemaire C., Cantineau R., Guillaume M., Plenevaux A., Christiaens L. (1991). Fluorine-18-Altanserin: A Radioligand for the Study of Serotonin Receptors with PET: Radiolabeling and in Vivo Biologic Behavior in Rats. J. Nucl. Med..

[145] Lundkvist C., Halldin C., Ginovart N., Nyberg S., Swahn C.-G., Carr A.A., Brunner F., Farde L. (1996). [^11^C]MDL 100907, a Radioligand for Selective Imaging of 5-HT_2A_ Receptors with Positron Emission Tomography. Life Sci..

[146] Meyer J.H., Kapur S., Houle S., DaSilva J., Owczarek B., Brown G.M., Wilson A.A., Kennedy S.H. (1999). Prefrontal Cortex 5-HT_2_ Receptors in Depression: An [^18^F]Setoperone PET Imaging Study. Am. J. Psychiatry.

[147] Rosales Martínez A., Rodríguez-García I., López-Martínez J.L. (2021). Divergent Strategy in Marine Tetracyclic Meroterpenoids Synthesis. Mar. Drugs.

[148] Rosales Martínez A., Rodríguez-Maecker R.N., Rodríguez-García I. (2023). Unifying the Synthesis of a Whole Family of Marine Meroterpenoids through a Biosynthetically Inspired Sequence of 1,2-Hydride and Methyl Shifts as Key Step. Mar. Drugs.

